# Modeling cell-specific dynamics and regulation of the common gamma chain cytokines

**DOI:** 10.1016/j.celrep.2021.109044

**Published:** 2021-04-27

**Authors:** Ali M. Farhat, Adam C. Weiner, Cori Posner, Zoe S. Kim, Brian Orcutt-Jahns, Scott M. Carlson, Aaron S. Meyer

**Affiliations:** 1 Department of Bioengineering, Jonsson Comprehensive Cancer Center, Eli and Edythe Broad Center of Regenerative Medicine and Stem Cell Research, University of California, Los Angeles, Los Angeles, CA 90024, USA; 2 Visterra, Inc., Waltham, MA 02451, USA; 3 These authors contributed equally; 4 Lead contact

## Abstract

The γ-chain receptor dimerizes with complexes of the cytokines interleukin-2 (IL-2), IL-4, IL-7, IL-9, IL-15, and IL-21 and their corresponding “private” receptors. These cytokines have existing uses and future potential as immune therapies because of their ability to regulate the abundance and function of specific immune cell populations. Here, we build a binding reaction model for the ligand-receptor interactions of common γ-chain cytokines, which includes receptor trafficking dynamics, enabling quantitative predictions of cell-type-specific response to natural and engineered cytokines. We then show that tensor factorization is a powerful tool to visualize changes in the input-output behavior of the family across time, cell types, ligands, and concentrations. These results present a more accurate model of ligand response validated across a panel of immune cell types as well as a general approach for generating interpretable guidelines for manipulation of cell-type-specific targeting of engineered ligands.

## In brief

Farhat et al. develop a mechanistic model of the common γ-chain receptor cytokines incorporating the structure of receptor-ligand interaction and trafficking. This model can predict the response to these cytokines, alone and in combination, and changes in binding affinity, enabling more rational cytokine engineering.

## Graphical Abstract

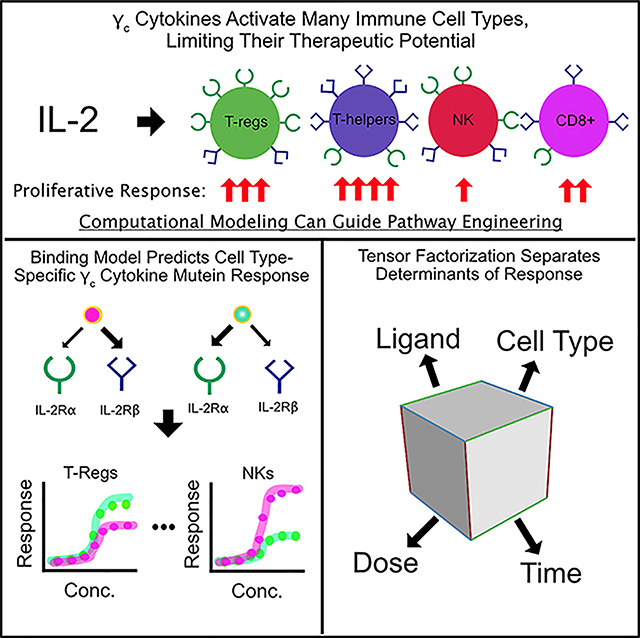

## INTRODUCTION

Cytokines are cell signaling proteins responsible for cellular communication within the immune system. The common γ-chain (γ_c_) receptor cytokines, including interleukin-2 (IL-2), IL-4, IL-7, IL-9, IL-15, and IL-21, are integral for modulating innate and adaptive immune responses. Therefore, they have existing uses and future potential as immune therapies (Leonard et al., 2019; Rochman et al., 2009). Each ligand binds to its specific private receptors before interacting with the common γ_c_ receptor to induce signaling (Walsh, 2010). γ_c_ receptor signaling induces lymphoproliferation, offering a mechanism for selectively expanding or repressing immune cell types (Amorosi et al., 2009; Vigliano et al., 2012). Consequently, loss-of-function or reduced-activity mutations in the γ_c_ receptor can cause severe combined immunodeficiency (SCID) because of insufficient T and natural killer (NK) cell maturation (Wang et al., 2011). Deletion or inactivating mutations in IL-2 or its private receptors leads to more selective effects, including diminished regulatory T cell (T_reg_) proliferation and loss of self-tolerance (Horak, 1995; Sharfe et al., 1997; Sharma et al., 2007). Deficiency in the IL-2 receptor IL-2Rα also causes hyperproliferation in CD8+ T cells but a diminished antigen response (Goudy et al., 2013). These examples show how γ_c_ receptor cytokines coordinate a dynamic balance of immune cell abundance and function.

The γ_c_ cytokines’ ability to regulate lymphocytes can affect solid and hematological tumors (Pulliam et al., 2016). IL-2 is an approved, effective therapy for metastatic melanoma, and the antitumor effects of IL-2 and IL-15 have been explored in combination with other treatments (Bentebibel et al., 2019; Zhu et al., 2015). Nonetheless, understanding these cytokines’ regulation is stymied by their complex binding and activation mechanism (Walsh, 2010). Any intervention imparts effects across multiple distinct cell populations, with each population having a unique response defined by its receptor expression (Cotari et al., 2013; Ring et al., 2012). These cytokines’ potency is largely limited by severe toxicity, such as deadly vascular leakage with IL-2 (Krieg et al., 2010). Finally, IL-2 and IL-15 are cleared rapidly renally and by receptor-mediated endocytosis, limiting their half-life *in vivo* (Bernett et al., 2017; Donohue and Rosenberg, 1983; Konrad et al., 1990).

To address the limitations of natural ligands, engineered proteins with potentially beneficial properties have been produced (Leonard et al., 2019). The most common approach has been to develop mutant ligands by modulating the binding kinetics of specific receptors (Berndt et al., 1994; Collins et al., 1988). For example, mutant IL-2 forms with a higher binding affinity for IL-2Rβ or reduced binding to IL-2Rα and induces greater cytotoxic T cell proliferation, antitumor responses, and proportionally less T_reg_ expansion (Bentebibel et al., 2019; Levin et al., 2012). This behavior can be understood through IL-2’s typical mode of action, in which T_reg_ cells are sensitized to IL-2 by expression of IL-2Rα (Ring et al., 2012). Bypassing this sensitization mechanism shifts cell specificity (Levin et al., 2012). Conversely, mutants skewed toward IL-2Rα over IL-2Rβ binding selectively expand T_reg_ cell populations over cytotoxic T cells and NK cells compared with native IL-2 (Bell et al., 2015; Peterson et al., 2018).

The therapeutic potential and complexity of this family make computational models especially valuable for rational engineering. Early attempts to mathematically model the synergy between IL-2 and IL-4 in B and T cells successfully identified a phenomenological model that could capture the synergy between the two cytokines (Burke et al., 1997). A cell population model has explained how T_reg_ cell IL-2 consumption suppresses effector T cell signaling (Feinerman et al., 2010). However, any model needs to incorporate the key regulatory features of a pathway to accurately predict cell response. With structural information that clarified the mechanism of cytokine binding, for example, a model of IL-4, IL-7, and IL-21 binding revealed pathway crosstalk depending on the relative γ_c_ receptor affinities (Gonnord et al., 2018). Nevertheless, these models have not accounted for endosomal trafficking and have not been constructed to model multiple immune cell types. The crucial role receptor-mediated endocytosis has been shown to play in signaling and drug delivery processes has led to development of many mathematical models incorporating its effects (Lao et al., 2007; Byun and Jung, 2020). IL-2 induces rapid endocytosis-mediated IL-2Rα and IL-2Rβ downregulation (Duprez et al., 1988; Ring et al., 2012), and trafficking is known to be a potent regulatory mechanism for all members of the γ_c_ family (Lamaze et al., 2001). Indeed, recent IL-15 engineering observed that attenuated cytokine potency can lead to a greater therapeutic effect via reduced receptor-mediated clearance (Bernett et al., 2017). Non-intuitive properties such as this can be better understood and optimized through models incorporating trafficking.

Here we assemble a predictive model and tools to visualize γ_c_ cytokine family regulation. We first built a family-wide mathematical model that incorporates binding and trafficking kinetics. This more comprehensive model allows us to investigate emergent behavior, such as competition between cytokines. This cytokine family is inherently highly dimensional, with multiple ligands, cognate receptors, and cells with distinct expression. Therefore, we use tensor factorization to visualize the family-wide regulation. This map helps us to identify how native or engineered ligands are targeted to specific immune cell populations based on their receptor expression levels. The methods used here can be used similarly in experimental and computational efforts of decoding other complex signaling pathways, such as Wnt, Hedgehog, Notch, and bone morphogenic protein (BMP)/transforming growth factor β (TGF-β) (Antebi et al., 2017a, 2017b; Eubelen et al., 2018; Li et al., 2018).

## RESULTS

### A model including trafficking captures IL-2 and IL-15 dose response and the effect of IL-2Rα expression

**Figure 1.**
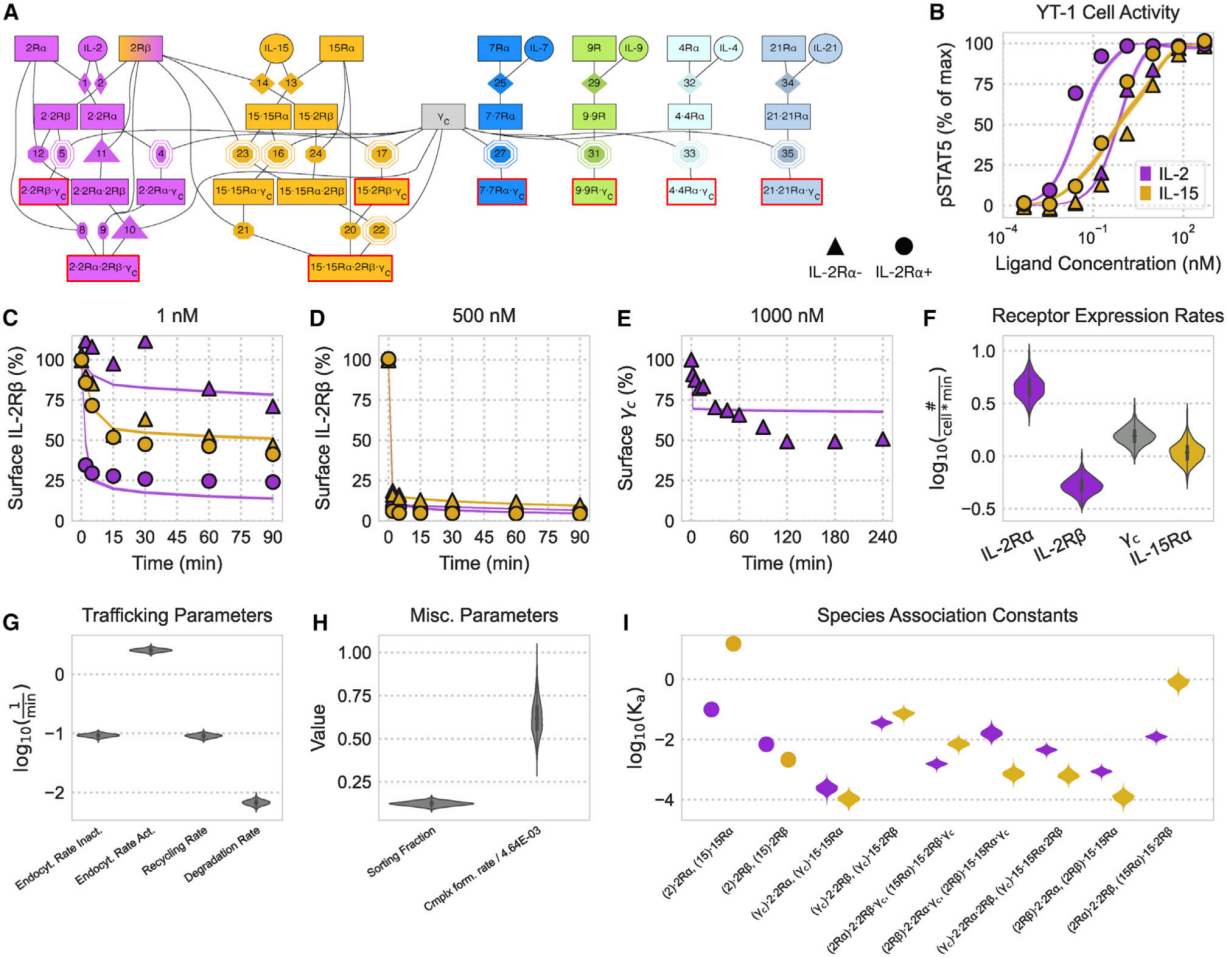
Unifying receptor binding and trafficking provides an accurate model of IL-2 and IL-15 response Experimental data were collected in previous studies (Mitra et al., 2015; Ring et al., 2012). (A) Schematic of all receptor (boxes)-ligand (circles) complexes and binding events. Active (pSTAT signaling; containing two signaling-competent receptors) complexes are outlined in red. Rate constants obtained from the literature, detailed balance, and fitting are denoted by diamonds, octagons, and octagons with a double outline, respectively. Rate constants that were measured experimentally relative to other rates are denoted by triangles. A scalar factor scales active receptor complexes to pSTAT predictions. See STAR Methods for full model equations. (B–E) Model fit to experimental results, represented by shaded regions and shapes respectively, for (B) pSTAT5 in YT-1 cells under various concentrations of ligand stimulation for 500 min and (C–E) the percent of initial IL-2Rβ (C and D) and γ_c_ (E) on the cell surface for various ligand stimulation concentrations. The 25%–75% and 10%–90% confidence intervals of the model’s fit are shaded dark and light, respectively. Note that only the 25%–75% interval is visible. (F–H) Posterior distributions after data fitting. The forward receptor dimerization rate k_fwd_ has units of *cell*3*#*^−1^×*min*^−1^, and the sorting fraction (f_sort_) is unitless. (I) Posterior distributions for the analogous association constants of IL-2 and IL-15. Association constants measured in the literature are represented by dots. Association constants are shown for species in parentheses complexing with the following species. K_a_s for (2)·2Rα, (15)·15Rα, (2)·2Rβ, and (15)·2Rβ have nanomolar units; all other K_a_s have units of *#*×*cell*^−1^. See also Figures S1 and S2.

To model how individual binding events give rise to cell response, we built a differential equation model representing the relevant binding and regulatory mechanisms in the γ_c_ receptor cytokine family (Figure 1A). The differential equations and corresponding rate parameters that define our model are described in the STAR Methods (Table 1). Binding interactions were modeled based on their known structural components and led to formation of receptor complexes capable of Janus kinase (JAK)/signal transducer and activator of transcription (STAT) signaling (Rochman et al., 2009). Endocytic trafficking of cell surface receptors is a critical mechanism of regulatory feedback (Basquin et al., 2013; Fallon and Lauffenburger, 2000; Fallon et al., 2000; Volkó et al., 2019). Therefore, we extended earlier modeling efforts by including trafficking of receptors and their complexes (Feinerman et al., 2010; Ring et al., 2012). We assumed that species trafficked into an endosomal compartment while continuing to produce JAK/STAT signaling and participating in binding events.

Rate parameters for IL-2 and IL-15 binding events were parameterized by previous experimental measurements and detailed balance or estimated by model fitting to existing experimental measurements (Figures 1B–1E). Fitting was performed to measurements of STAT5 phosphorylation and surface IL-2Rβ/γ_c_, upon IL-2 or IL-15 stimulation, in wild-type YT-1 human NK cells or YT-1 cells selected for expression of IL-2Rα. The experimental data were collected from previous studies (Mitra et al., 2015; Ring et al., 2012). The posterior parameter distributions from these fits (Figure 1F–1I) were plugged back into our model and showed quantitative agreement with the data, including differential sensitivity with IL-2Rα expression (Figures 1B–1E; Mitra et al., 2015; Ring et al., 2012). To evaluate the effect of including trafficking, we fit a version of the model without trafficking to the same pSTAT5 measurements. Surprisingly, the model without trafficking was able to fit the data equally well with small changes to some inferred rate constants (Figure S1). Although the model with trafficking inferred cell receptor expression of ~1 receptor/cell/min, corresponding to 500–5,000 receptors/cell, the model without trafficking inferred that YT-1 cells have receptor abundances of 1–10/cell. We elected to use the model including trafficking for the duration of the study because γ_c_ receptors have known trafficking regulation. We also show that endocytic signaling can uniquely affect the cell-type-specific response to γ_c_ cytokines (Figure 6C) and that trafficking improves model correspondence to our validation measurements (Figures S5 and S6). Depletion of surface IL-2Rβ and γ_c_ occurs through rapid endocytosis of active complexes, and indeed, depletion occurred faster at higher cytokine doses (Figures 1C–1E). Correspondingly, active complex internalization was inferred to be ~10× greater than that for inactive species (Figure 1G). These data suggest that trafficking and binding can be integrated in a model of IL-2 and IL-15 signaling response.

Because IL-2 and IL-15 drive formation of analogous active complexes with IL-2Rβ, γ_c_, and a signaling-deficient high-affinity receptor (IL-2Rα/IL-15Rα), comparing their inferred binding rates gave insight into how IL-2 and IL-15 differ from one another (Figure 1I). The two ligands have nearly the same direct binding affinity to IL-2Rβ; however, IL-15 has a higher affinity than IL-2 for its α chain. Consequently, IL-15’s complexes were inferred to more readily dimerize with a free α chain than IL-2’s complexes. The other dimerization affinities were generally similar between IL-2 and IL-15. The unbinding rate constants were consistent with the literature indicating that IL-2 has a higher affinity for IL-2Rβ when bound to its α chain (Spangler et al., 2015). A model of IL-2 and IL-15 incorporating trafficking is consistent with known biophysical and cell response measurements.

### The family model correctly captures IL-4/IL-7 dose response and cross-inhibition

To further test our model incorporating trafficking, we evaluated its performance in a series of experiments involving IL-4 and IL-7. IL-2 and IL-15 involve the same signaling-competent receptors, and so the signaling activity of each cytokine cannot be distinguished. IL-4 and IL-7 activity, in contrast, can be distinguished when both cytokines are co-administered to cells by measuring STAT6 and STAT5 phosphorylation, respectively (Leonard et al., 2019). Using this phenomenon, we explored previously published cross-inhibition data where IL-4 and IL-7 doses were administered to human peripheral blood mononuclear cell (PBMC)-derived CD4^+^TCR^+^CCR7^high^ T cells individually and together (Gonnord et al., 2018).

**Figure 2.**
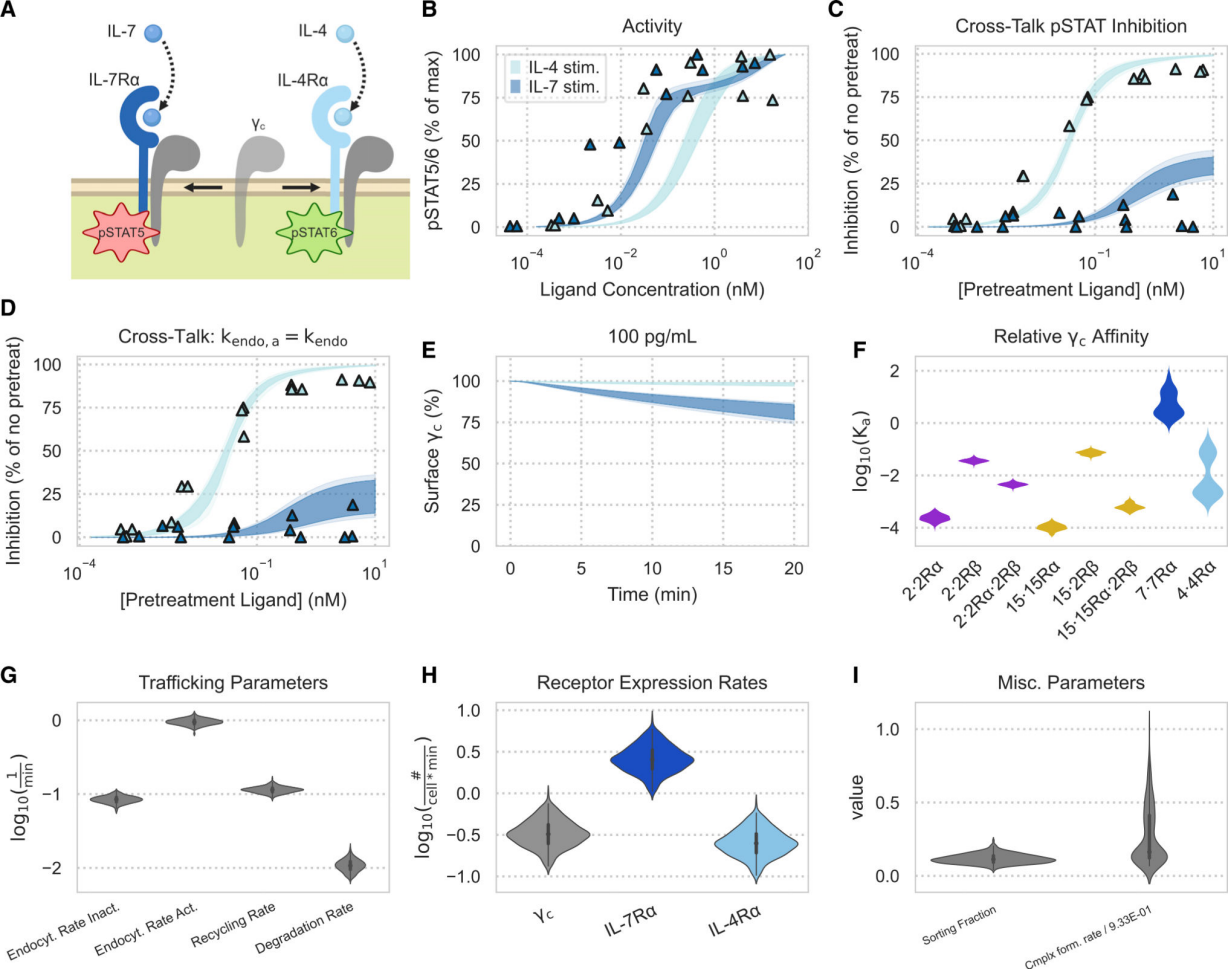
A reaction model captures cytokine-cytokine interactions Experimental data were collected in previous studies (Gonnord et al., 2018). (A) Schematic of IL-4 and IL-7 receptor complexes competing for γ_c_ and generating distinct pSTAT signals. (B and C) Model fits to experimental data. Experimental measurements are denoted by triangles. Shaded areas represent the 25%–75% and 10%–90% confidence intervals of the model fit. pSTAT5 and pSTAT6 were measured for IL-7 and IL-4 experiments, respectively. (B) Single-cytokine pSTAT dose-response measurements for 10 min of exposure to IL-4 and IL-7. The experiment was performed in duplicate (n = 2). (C) Percent inhibition of the second cytokine’s pSTAT response in a dual-cytokine dose-response experiment. Human PBMC-derived T cells (CD4^+^TCR^+^CCR7^high^) were pretreated with various concentrations of one cytokine for 10 min before being stimulated with a fixed concentration (2 pM IL-7 or 6.25 pM IL-4) of the other cytokine for an additional 10 min. (D) Model inference for percent inhibition of the second cytokine’s pSTAT response in a dual-cytokine dose-response experiment after setting active species to be endocytosed at the same rate as inactive species. (C and D) Experiments were performed in triplicate (n = 3). (E) Model predictions for the percentage of γ_c_ on the cell surface when exposed to 100 pg/mL of IL-7 or IL-4 for 20 min. (F) Violin plot of K_a_ values (units of *#*3*cell*^−1^) for complexes with γ_c_ obtained via the posterior distribution of the forward and reverse binding rate parameters. (G–I) Posterior parameter distributions from fitting to data. The forward dimerization rate k_fwd_ has units of *cell*3*#*^−1^3*min*^−1^.

Using surface abundance measurements of IL-4Rα, IL-7Rα, and γ_c_, we applied a steady-state assumption in the absence of ligand to solve each receptor expression rate (Gonnord et al., 2018). Our model fit single and dual cytokine dose-response data with reasonable accuracy. Fits to the IL-4 and IL-7 dose response had systematic deviation toward higher half maximal effective concentration (EC_50_) values (Figure 2B), but the model captured the difference in response between IL-4 and IL-7 as well as the effects of cross-inhibition (Figures 2B–2C). Some systematic error in the model can be expected, given our focus on receptor binding features and subsequent choice to not model the JAK-STAT pathway in total. The fitting process identifiably constrained the reaction rates and trafficking parameters (Figure 2F–2I). Although surface abundance was constrained, the receptor expression rates still formed distributions dependent on trafficking parameters (Figures 2G–2I).

The experimental data and model fits showed that IL-7 inhibited IL-4 signaling response more than vice versa (Figure 2C; Gonnord et al., 2018). Consistent with the experimentally derived mechanism (Gonnord et al., 2018), this inhibitory behavior was explained by the competition of ligand·α chain complexes for the common γ_c_. The inferred association constant (K_a_) value of this dimerization process for IL-7 was larger than the K_a_ value for IL-4, indicating that there was tighter dimerization of IL-7·IL-7Rα to γ_c_ than of IL-4·IL-4Rα to γ_c_ (Figure 2F). The competition for γ_c_ was determined to play a larger role in signaling inhibition than receptor internalization because our model showed that the same inhibitory relationships hold when active complexes were set to internalize at the same rate as other species (Figure 2D). Internalization was also dismissed because much of the γ_c_ remained on the cell surface after ligand stimulation in model simulations and experimental measurements (Figure 2E; Gonnord et al., 2018).

### Tensor factorization maps the γ_c_ family response space

**Figure 3.**
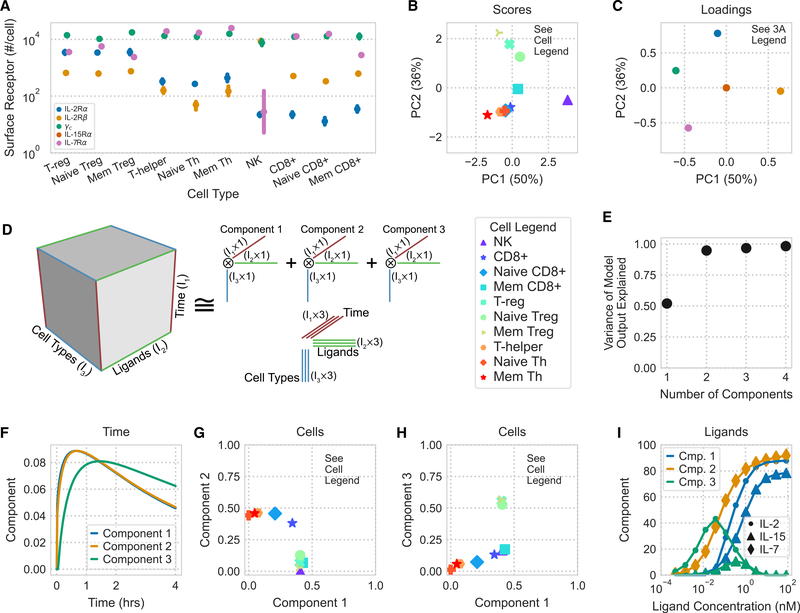
Tensor factorization map model-predicted cytokine responses (A) Measured receptor abundance for 10 PBMC-derived subpopulations gathered from a single donor, measured by flow cytometry. Points and error bars show geometric mean and standard error, respectively (n = 4). Error bars for some points are too small to display. (B and C) PCA scores (B) and loading (C) of receptor abundance. Axis label percentages indicate percent variance explained. (D) Schematic representation of CP decomposition. Model predictions are arranged in a cube depending on the time, ligand treatment, and cell type being modeled. CP decomposition then helps to visualize this space. (E) Percent variance reconstructed (R2X) versus the number of components used in non-negative CP decomposition. (F–I) Component values versus time (F), cell type (G and H), and ligand stimulation (I). The variation explained by each component is the product of the component’s time, ligand, and cell type factorization. Ligand components with only negligible values (<15% max) are not shown. See also Figures S3 and S4.

Because response to ligand is mostly defined by receptor expression, we quantitatively profiled the abundance of each IL-2, IL-15, and IL-7 receptor across 10 PBMC subpopulations (Figure 3A). PBMCs gathered from a single donor were stained using receptor-specific fluorescent antibodies and analyzed by flow cytometry; their subpopulations were separated using canonical markers (Figure S3; Table S1). These data recapitulated known variation in these receptors, including high IL-7Rα or IL-2Rα expression in helper and T_reg_ cells, respectively (Hassan and Reen, 1998; Rochman et al., 2009). Principal-component analysis (PCA) helped to further visualize variation in these receptor abundance data. The 10 PBMC cell types were mapped in the scores plot (Figure 3B) using two principal components, each of which was defined by a linear combination of the cell’s receptor expression abundance, as described in the loadings plot (Figure 3C). Principal component 1, which explained 50% of the receptor expression data’s variance, most prominently separated NK cells from all others because of their distinct receptor expression, featuring high levels of IL-2Rβ and relatively lower levels of γ_c_ compared with other cell types, which are strongly correlated positively and negatively with principal component 1, respectively. Principal component 2, which explained 36% of the receptor expression data’s variance, then separated effector and T_reg_ cell populations based on their high IL-7Rα or IL-2Rα abundance, respectively. PCA also helped to highlight the subtly higher γ_c_ levels in T_reg_ cells and the slightly more T_reg_ cell-like profile of memory CD8+ cells.

Even with an accurate model, exploring how dynamic responses vary across responding cell types and ligand treatments remains challenging. Considering only a single time point, cell type, or ligand concentration provides only a slice of the picture. Therefore, we sought to apply factorization as a method to globally visualize ligand response.

To build a tensor of model predictions, we assembled simulation predictions across cell types, ligand conditions, and time. This three-dimensional (time, cell type, and ligand) tensor was then decomposed with non-negative canonical polyadic (CP) decomposition (Figure 3D). We selected three components during decomposition because this number captured 95% of the variance in our original data tensor (Figure 3E). To show the relationships among the tensor’s three dimensions, the component plots of each dimension were plotted alongside each other.

CP decomposition can be interpreted by matching a single component’s effects across factor plots for each dimension, allowing us to interpret its relationship to time, to a profile of cell responses, and a pattern of stimulation conditions (Figures 3F–3I). For example, component 2 is greatest at roughly 50 min (Figure 3F) for helper and CD8+ T cells (Figure 3G) and occurs almost exclusively with IL-7 stimulation (Figure 3I). This indicates that this variation in the data occurs with IL-7 stimulation, leads to a response in helper and CD8+ T cells, and peaks at 50 min. In this way, different contributory factors in cell response are separated.

All components showed similar variation with time, peaking quickly and then decreasing after roughly 50 min (Figure 3F). This can be understood as two phases: one dominated by receptor activation and a second with trafficking-mediated downregulation of the receptors (Figure 1). Comparing the cells and ligand decomposition plots showed the expected effects. IL-7 response was separated by component 2, which showed a dose-dependent increase, and correlated with IL-7Rα expression levels (Figures 3A, 3G, and 3I). Interestingly, IL-2/15 response separated by concentration rather than ligand (Figure 3I). Low concentrations of IL-2 were represented by component 3, and preferentially activated T_reg_ over effector T cells (Figures 3H and 3I). High concentrations of IL-2/15 were represented by component 1 and similarly activated effector and T_reg_ cells (Figures 3G and 3I). This known dichotomy occurs through higher IL-2Rα expression in T_reg_ cells (Figure 3A). Importantly, although PCA can help to distinguish cells based on distinct receptor expression profiles, cells separated differently based on their predicted ligand stimulation response (Figures 3B, 3G, and 3H). This demonstrates the unique benefit of tensor- and model-based factorization to distinguish cells based on their predicted response profiles.

Other tensor decomposition methods exist and can be applied similarly to visualize response. For example, non-negative Tucker decomposition relaxes CP decomposition by employing a core tensor that provides interaction terms between components (Figure S4; Tucker, 1966). However, this flexibility comes at the cost of interpretability because visualizing the core tensor’s effect is challenging. In total, factorization methods are effective means of visualizing the high-dimensional regulation of complex receptor families and separating the influence of time, ligand stimulation, and receptor expression.

### An accurately predicted response across a panel of PBMC-derived cell types

**Figure 4.**
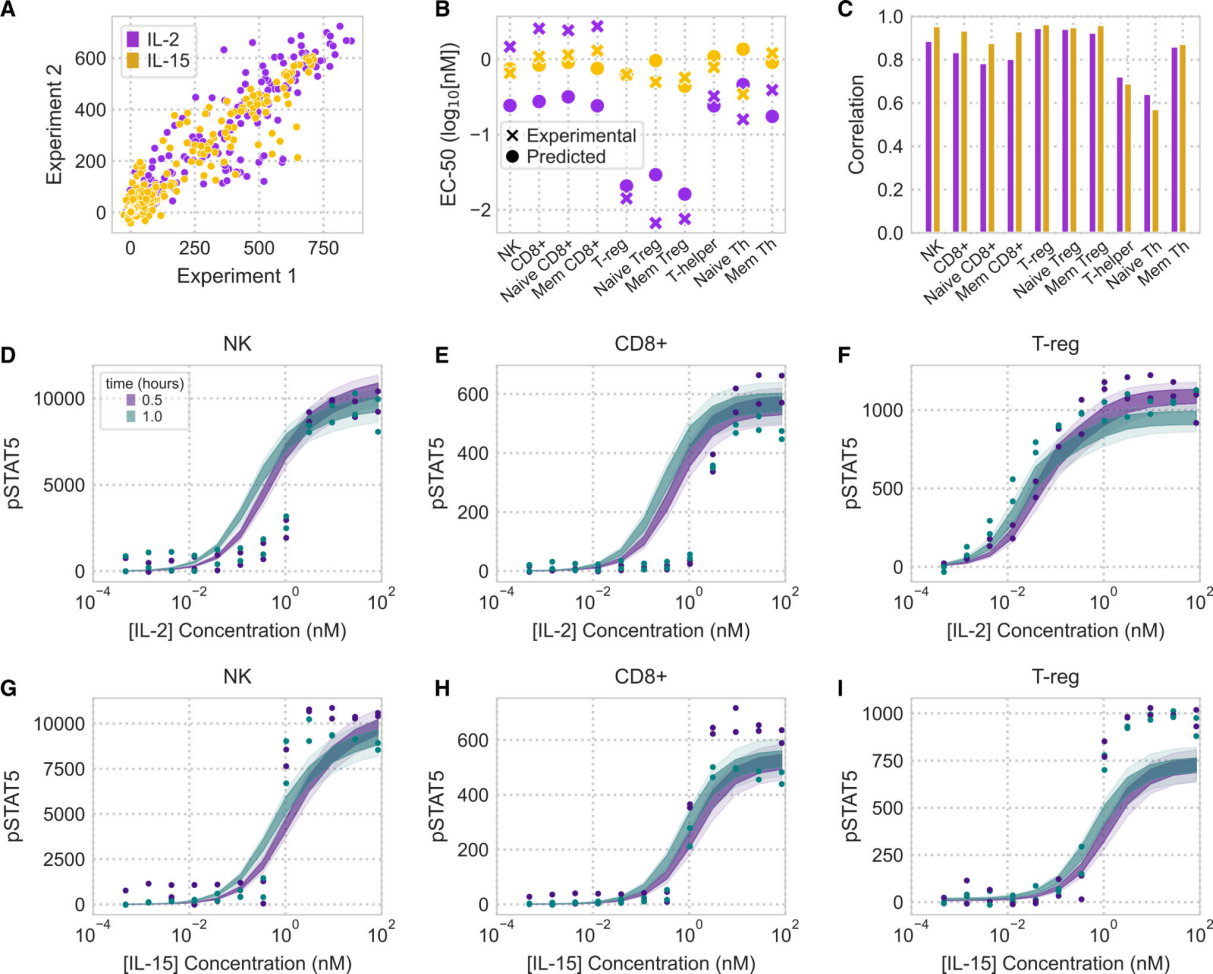
The model accurately predicts cell-type-specific response across a panel of PBMC-derived cell types (A) Comparison of two experimental replicates measuring the pSTAT5 response of PBMC-derived cells to cytokine stimulation. Points represent flow cytometry measurements from each cell type to a dose response of IL-2 or IL-15 at multiple time points and have units of median fluorescence intensity. (B) Experimentally derived and model-predicted EC_50_ values of dose response across IL-2/15 and all 10 cell types. EC_50_ values are shown for the 1-h time point. (C) Pearson correlation coefficients between model prediction and experimental measurements for all 10 cell populations (full data are shown in Figure S5). (D–I) pSTAT5 response to IL-2 (D–F) and IL-15 (G–I) dose responses in NK, CD8+, and T_reg_ cells. Experiments were performed in duplicate (n = 2). See also Figure S5.

We evaluated whether our model accurately predicts cell-type-specific differences in ligand response by comparing its predictions for IL-2/15 responses across a panel of 10 PBMC-derived cell populations. We measured and used our model to predict PBMC response to cytokine stimulation at 12 concentrations (0.5 pM–84 nM) and 4 time points (30 min, 1 h, 2 h, and 4 h). Individual cell types displayed reproducible responses to IL-2/15 treatment (Figure 4A). Overall, our model predictions of ligand pSTAT5 response closely matched experimental measurements (Figure 4; Figure S5). The differences between cell types largely matched known differences in cytokine response. For example, T_reg_ cells were markedly sensitive to IL-2 (Figures 4B and 4F), but not IL-15 (Figures 4B and 4I), at low concentrations of the cytokine (Bell et al., 2015; Peterson et al., 2018). Small amounts of IL-2Rα in helper T cells (Figure 3A) partially sensitized them to IL-2 (Figure 4B; Figures S5H–S5J). The model was also able to partly predict downregulation of pSTAT response at 2 and 4 h by including receptor trafficking (Figure S5). Although our model was slightly less accurate in predicting T helper response to cytokine stimulation, it was able to broadly and accurately capture differences in sensitivity and response across all the cell populations (Figure 4C).

To further evaluate the importance of receptor trafficking, we also predicted PBMC response using our model without trafficking included (Figure S1). This model completely failed to predict PBMC cytokine responses across all populations (Figure S6). We expect this arose from the large difference in inferred receptor abundance when fitting the two models. The model without trafficking required very small amounts of receptor abundance to fit the YT-1 responses and therefore failed with the PBMC case, where we experimentally measured the receptor amounts. This difference in performance clearly demonstrates that incorporating trafficking is necessary to develop a model that generalizes to new contexts.

Although the model accurately predicted experimentally measured responses overall, we noticed some larger discrepancies specifically at high ligand concentrations and after 2 h in specific cell populations (Figure 4; Figure S5). For example, although CD8+ cells almost exactly matched model predictions at 1 h, by 4 h we experimentally observed a biphasic response with respect to IL-2 concentration and a plateau with IL-15 that decreased over time. This decrease in signaling was most pronounced with CD8+ cells but could be observed to lesser extents in some other cell populations such as NK cells (Figure S5). We hypothesize two possible explanations for this discrepancy. First, CD8+ populations are known to proteolytically shed IL-2Rα in an activity-responsive manner (Junghans and Waldmann, 1996). Second, our model does not encompass the JAK-STAT pathway, whose components surely influence dynamic response (Kuwabara et al., 2016). Our model also had a quantitative difference from experimental results for the pSTAT5 EC_50_ variation between effector and regulatory cells (Figures 4B, 4D, and 4E). However, overall, the model presented here remains useful for exploring the determinants of cell-type-specific response, which originate at the receptor expression profile on the cell surface. The broad experimental profiling here will also enable future model refinement.

### Tensor factorization of experimental measurements distinguishes the cell-type-specific response

**Figure 5.**
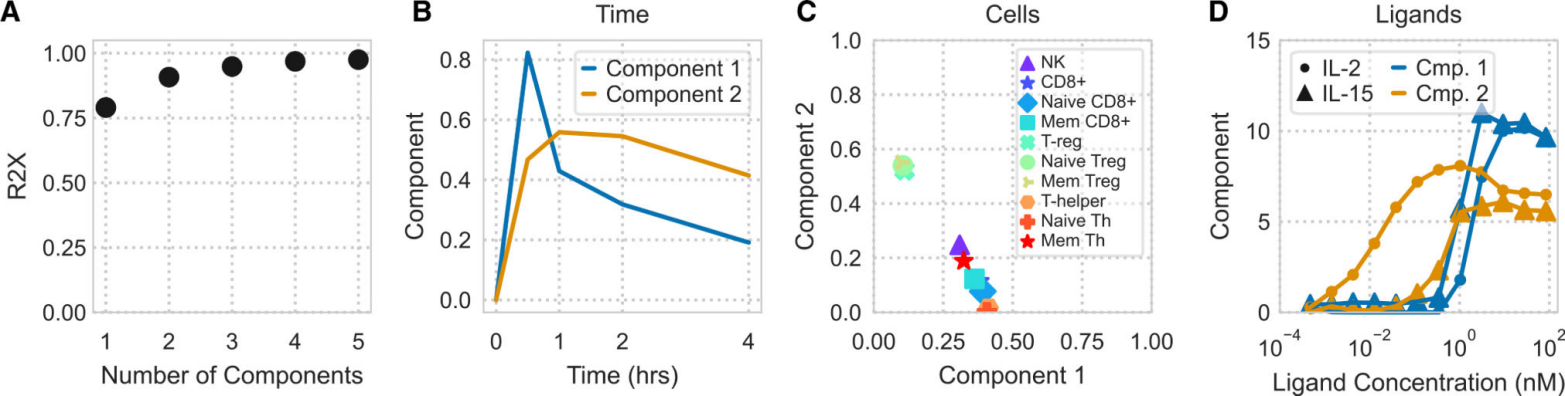
Non-negative CP decomposition applied to experimental pSTAT5 measurements (A) R2X of non-negative CP decomposition versus number of components used. (B–D) Decomposition plots with respect to time (B), cell type (C), or ligand treatment (D). See also Figure S4.

Given that tensor factorization helped to visualize model predictions of IL-2, IL-7, and IL-15 response, we wished to evaluate whether it could similarly help visualize experimental measurements. We structured our experimental pSTAT5 measurements in an identical format as the model simulation tensor (Figure 3). Two components explained roughly 90% of the variance in the original data (Figure 5A), which we then interpreted using similar factor plots (Figures 5B–5D).

Interestingly, as seen with the model prediction factorization, factors were distinguished by their concentration more than being tied to a specific ligand (Figure 5D). Component 2 increases with low concentrations of IL-2, whereas component 1 only increases at high concentrations of either ligand. As expected, effector and T_reg_ cells are most strongly associated with components 1 and 2, respectively, matching their known dose-response profiles (Figure 4). However, component 2 is also distinct from component 1 in its sustained signaling (Figure 5B; Figure S5). This can be expected from rapid endocytosis-mediated downregulation of IL-2Rβ at high IL-2/−15 concentrations (Figure 1). Thus, tensor factorization helps to separate these differences in dose- and cell-type-specific responses. Furthermore, there was clear, quantitative correspondence between the model and experimental factorization. For example, both components from the experimental measurement factorization (Figure 5C) correlated strongly in their cell type weighting with their analogous pairs in the model factorization (cosine similarity of 0.98 and 0.89; Figure 3H).

### The model accurately captures the cell-type-specific response to IL-2 muteins

**Figure 6.**
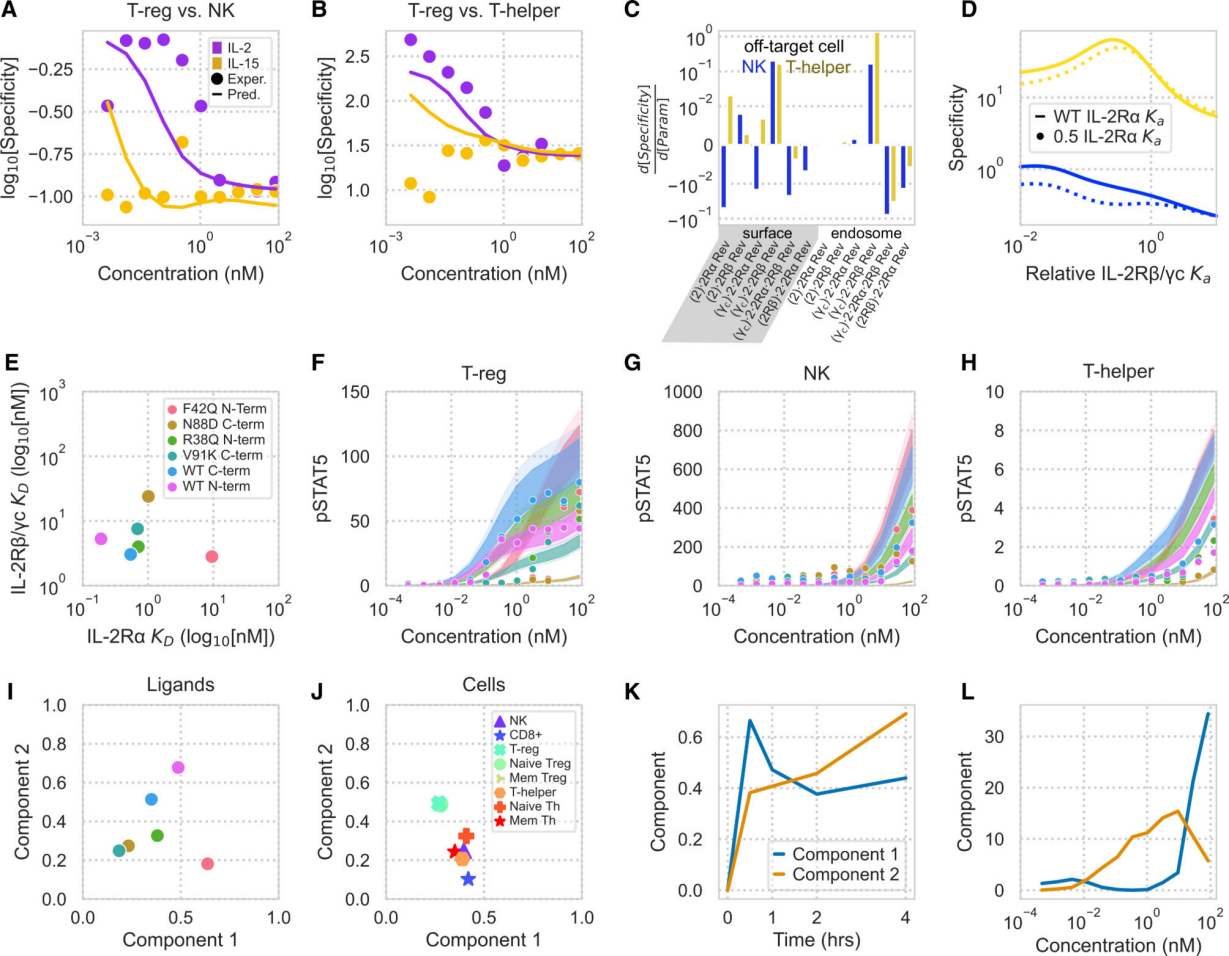
Model and tensor factorization predicts and decodes cell-type-specific responses to IL-2 muteins (A and B) Predicted and measured T_reg_ cell signaling specificity compared with NK (A) and T helper (B) cells at 1 h. Specificity is defined here as the ratio of two cell types’ pSTAT5. Experimental measures are average of two flow cytometry replicates (n = 2). (C) Partial derivatives of T_reg_ cell signaling specificity compared with NK and T helper cells with respect to each surface and endosomal reverse binding rate constant. (D) T_reg_ signaling specificity with respect to NK and T helper cells as a function of IL-2Rβ/γc binding affinity for ligands with wild-type and reduced IL-2Rα affinity. (C and D) Specificity values are shown for cells exposed to 38 pM of cytokine for 1 h. (E) IL-2Rα and IL-2Rβ/γ_c_ dissociation constants for our panel of IL-2 muteins, determined using bio-layer interferometry. (F–H) Predicted versus experimental immune cell responses to IL-2 muteins for T_reg_ cells (F), NK cells (G), and T-helper cells (H) for 1-h stimulation. Dots represent experimental flow cytometry measurements, and shaded regions represent the 10%–90% confidence interval for model predictions. Mutein stimulants are denoted by color. (I–L) Tensor factorization of experimentally measured cellular signaling values for IL-2 muteins. Shown are component values versus ligand (I), cell type (J), time (K), and cytokine concentration (L). See also Figures S7 and S8.

Using the model, we sought to identify strategies for selectively targeting T_reg_ cells. To quantify the effectiveness of selectively activating T_reg_ cells, we defined a specificity metric as the normalized pSTAT5 response of T_reg_ cells divided by the pSTAT5 response of T helper or NK cells. As expected, the model prediction and experimental values of this specificity increased with lower concentrations of IL-2 and had a lesser concentration-dependent relationship with IL-15 (Figures 6A and 6B). Our model was unable to quantitatively predict the specificity of T_reg_ cell signaling with respect to T helper cells, particularly for IL-15 stimulation. However, it was able to recapitulate the relationship of the quantity with IL-2 stimulation. With this quantity, we then examined the sensitivity of the specificity metric with respect to surface and endosomal binding. Increasing the dissociation rate of IL-2 from IL-2Rβ/γ_c_, particularly in the endosome, provided the largest and most consistent specificity increase (Figure 6C). Changes in endosomal binding rates have been shown to have important effects on a protein therapy’s half-life (Sarkar et al., 2002). To the extent this binding can be manipulated separately, the model indicates that it might help to improve specificity as well. Although IL-2Rβ/γ_c_ affinity was identified as most sensitive, the model predicted that ligands with reduced IL-2Rα affinity had decreased T_reg_ cell specificity regardless of their IL-2Rβ/γ_c_ affinity (Figure 6D). Therefore, reducing IL-2Rβ/γ_c_ affinity can help modulate the potency of these cytokines, but maintaining IL-2Rα affinity is still critical. These results demonstrate this model’s ability to predict immune cell response to wild-type or engineered cytokines, particularly for engineering cell-specific responses.

To evaluate these predictions, we measured the PBMC response to several Fc-fused IL-2 monomers. Wild-type and mutant forms of IL-2 were produced as fusions with a monomeric human antibody Fc domain. Targeted mutations were introduced to IL-2 in regions known to be instrumental for IL-2Rα or IL-2Rβ/γ_c_ binding. In particular, mutations at V91 and N88 are present in molecules being developed to treat autoimmune disease through selective IL-2 signaling in T_reg_ cells (Peterson et al., 2018, Ghelani et al., 2020, Gavin et al., 2017).

Cytokines are often Fc fused to increase the drug’s *in vivo* half-life and can be placed in either orientation. We quantified the effect of our engineered mutations and Fc fusion on IL-2Rα and IL-2Rβ/γ_c_ binding kinetics using bio-layer inferometry (Figure S7). Surprisingly, we found that Fc fusion to the N terminus selectively lowered IL-2Rβ/γ_c_ affinity, whereas fusion to the C terminus selectively lowered IL-2Rα affinity (Data S1; Figure 6E). Therefore, Fc fusion can have either complementary or counter-productive effects on mutation-mediated changes in receptor affinity, and affinity must be assessed in a clinical format. The observed changes in receptor-ligand kinetics caused by Fc-fusion were assessed for ligands fused using a 20-amino-acid linker; linkers of different lengths or flexibility likely also affect cytokine binding kinetics.

Using these altered affinities, we were able to accurately predict the cell-type-specific pSTAT5 response to our modified ligands (Figure S8; Figures 6F–6H). The model widely captured the cell-type-specific response to the muteins and especially the signaling response in the first 2 h. However, accuracy varied according to ligand and cell type and was noticeably reduced for NK cells and T_reg_ cell variants at higher concentrations and in predicting most responses to N88D. The model’s inaccuracy in predicting the N88D response is potentially to be expected because the N88D affinity for IL-2Rα and IL-2Rβ/γ_c_ is among the most drastically divergent from the wild-type IL-2 and IL-15 responses to which the model was fit (Figure 6E). Ligands with decreased IL-2Rα or IL-2Rβ/γ_c_ affinity had a decreased T_reg_ or T helper cell pSTAT5 response, respectively, as expected. As before, visualizing the effect of altered binding kinetics on cellular response is complicated by the contributions of cell type, concentration, and time (Figure 3). To visualize our results, we performed tensor factorization using the experimentally determined pSTAT5 response of PBMCs exposed to wild-type and modified IL-2 ligands (Figures 6I–6L). Two components explained 80% of the variance in the new combined data tensor. The two components matched those patterns from the model (Figures 3F–3I) and wild-type cytokines (Figure 5), with separation by cell type (Figure 6J) and concentration (Figure 6L) rather than ligand identity (Figure 6I) and a more sustained response by the T_reg_ cell-specific component (Figure 6K). Among the ligands, wild-type N-terminally conjugated IL-2 was the most potent inducer of T_reg_ cell response, as shown by its strong component 2 weighting (Figures 6I and 6J). The difference in signaling with Fc fusion orientation is likely due to the opposing effects on the cytokine’s IL-2Rα affinity (Figure 6I) because these different responses were matched by the model (Figure 6F).

## DISCUSSION

Here we built a mass action kinetic binding model for the common γ_c_ receptor family and used factorization methods to explore its cell-type-dependent behavior. This approach provided insights into its high-dimensional regulation. Our binding reaction model combined the structure of ligand interaction with endosomal trafficking, which allowed us to accurately model response (Figure 1). After fitting our model to previously published cytokine response data, we were able to predict IL-2, IL-2 mutein, and IL-15 response across a wide panel of PBMC-derived cell types (Figure 4; Figure S5). Mass action models can help to explain counterintuitive features of ligand response and identify specific strategies for optimizing therapeutically desired properties (Haugh, 2004; Meyer et al., 2015). In the case of the γ_c_ receptor cytokines, a therapeutic goal has been to specifically modulate subpopulations of cells based on their unique receptor expression profiles (Bell et al., 2015; Bentebibel et al., 2019; Levin et al., 2012; Peterson et al., 2018). To visualize these possibilities, we employed tensor factorization to map the signaling response space. This map provided a clearer picture of differential responsiveness between ligands, with selective and increased signaling for certain cells and ligands (Figures 5 and 6). For example, we could clearly identify the selectivity of IL-7 for T helper cells and low concentrations of IL-2 for T_reg_ cells (Figure 3).

The model described here serves as an effective tool for cell-type-selective rational cytokine design. In addition to the natural ligands, many cytokine muteins have been designed with altered binding affinities for specific receptors (Berndt et al., 1994; Collins et al., 1988). Our model serves as a computational tool for comparing these muteins as immunotherapeutic drugs that selectively activate certain cell populations. For example, our model helped to identify that high IL-2Rα affinity is essential to preserve T_reg_ cell specificity regardless of the affinity for IL-2Rβ/γ_c_ (Figure 6). The orientation of Fc fusion can significantly influence receptor affinity (including reducing IL-2Rα affinity), and so this step of drug design needs to be incorporated into ligand optimization (Figure 6E). Incorporating trafficking with the binding events of the cytokines allowed us to distinguish surface and endosomal binding, which is an unexplored axis for further engineering cell-specific responses. Indeed, endosomal IL-2Rα affinity is predicted to be more critical for T_reg_ cell specificity than binding on the surface, which agrees with the distinct temporal profiles of ligand response between cell types on the time-scale of trafficking (Figures 6C and 6K).

Models incorporating the full panel of responding cell populations will enable further refinement of these engineered ligands (León et al., 2013). IL-2 and IL-15 have extremely short half-lives *in vivo*, in part because of endocytosis-mediated clearance (Bernett et al., 2017; Konrad et al., 1990). Including endocytic trafficking of ligands will enable future work modeling ligand clearance *in vitro* and *in vivo*. Changes in receptor binding may therefore be selected based on optimized selectivity and pharmacokinetic properties. Although cell types were defined here by their average receptor expression, cell-to-cell variability within these populations leads to variation in stimulus response (Cotari et al., 2013). Incorporating single-cell variation will provide a more complete picture of population response and may help to further refine cell type selectivity.

Although the model was able to capture many of the overall differences and dynamics in cytokine response between cell populations and engineered ligands, we noted some systematic errors. In particular, predictions were generally worse for helper T cells (Figure 4C), longer and higher-concentration treatments (Figure S5), and engineered muteins with the largest changes in their receptor binding kinetics (Figure S8; N88D). We expect that there are three explanations for these errors that provide opportunities for further model refinement. First, we set a high bar for performance of the model by only fitting to cell line measurements and then trying to predict PBMC response as our validation. Any systematic differences between the YT-1 cell line and primary cultures would show up as an error in our model, and directly training the model on PBMC responses would reveal these. Second, we treat populations as overall averages, when cell-to-cell variation certainly exists (Cotari et al., 2013). As described above, modeling the variation in these populations could help correct for skewed responses that arise because of this heterogeneity. Finally, we elected to only model receptor-level regulatory events because these are most available for therapeutic engineering. However, the JAK-STAT pathway is dynamically regulated and certainly contributes to our measured responses (Kuwabara et al., 2016). Incorporating this pathway is sure to further improve our model’s correspondence to the data. Each of these improvements will, in turn, reveal other useful points to engineer this pathway.

Receptor families with many receptors and ligands are often made up of a dense web of connections, making the role of individual components non-intuitive (Antebi et al., 2017b; Eubelen et al., 2018). Interconnected, cross-reactive components may have evolved as a tradeoff between transmitting ligand-mediated information and expanding the repertoire of cell-surface proteins (Komorowski and Tawfik, 2019). The methods detailed in this paper can be applied to many signaling systems characterized by pleiotropy and high dimensionality. The combination of dynamic, mechanistic models and statistical exploration methods is particularly powerful to provide actionable directions for how to optimize therapeutic response. Detailed biophysical and structural characterization, animal disease models, and evidence from human genetic studies make this engineering possible for therapeutically targeting other complex signaling pathways, including FcγR, Wnt, Hedgehog, Notch, and BMP/TGF-β (Antebi et al., 2017a, 2017b; Eubelen et al., 2018; Li et al., 2018; Robinett et al., 2018).

## STAR★METHODS

### RESOURCE AVAILABILITY

#### Lead contact

Further information and requests for resources and reagents should be directed to and will be fulfilled by the Lead Contact, Aaron Meyer (a@asmlab.org).

#### Materials availability

Materials generated in this study are available upon reasonable request from the lead contact.

#### Data and code availability

All datasets generated during and/or analyzed during the current study and all custom scripts and functions generated or used during the current study are available from https://github.com/meyer-lab/gc-cytokines.

### EXPERIMENTAL MODEL AND SUBJECT DETAILS

#### Cell lines

Cryopreserved PBMCs (ATCC, PCS-800–011, lot#81115172) were harvested from a single adult human subject.

### METHOD DETAILS

#### Base model

Cytokine (IL-2, −4, −7, −9, −15, & −21) binding to receptors was modeled using ordinary differential equations (ODEs). IL-2 and −15 each had two private receptors, one being a signaling-deficient α chain (IL-2Rα & −15Rα) and the other being signaling-competent IL-2Rβ. The other four cytokines each had one signaling-competent private receptor (IL-7Rα, −9R, −4Rα, & −21Rα). JAK-STAT signaling is initiated when JAK-binding motifs are brought together. JAK binding sites are found on the intracellular regions of the γ_c_, IL-2Rβ, IL-4Rα, IL-7Rα, IL-9R, and IL-21Rα receptors; therefore, all complexes which contained two signaling-competent receptors were deemed to be active species. Ligands were assumed to first bind a receptor other than γ_c_ and then can dimerize with other receptors or γ_c_ thereafter. Direct binding of ligand to γ_c_ was not included due to its very weak or absent binding (Voss et al., 1993). Our model’s output was defined by the number of active signaling complexes; experimental STAT phosphorylation measurements were scaled to model predictions by use of a fit scalar factor.

In addition to binding interactions, our model incorporated receptor-ligand trafficking. Receptor synthesis was assumed to occur at a constant rate. The endocytosis rate was defined separately for active (k_endo,a_) and inactive (k_endo_) receptors. f_sort_ defined the fraction of endosomal species that ultimately traffic to the lysosome; active species in the endosome had a sorting fraction of 1.0. All endosomal species not sent to lysosomes were recycled back to the cell surface. The lysosomal degradation and recycling rate constants were defined as k_deg_ and k_rec_, respectively. We assumed no autocrine ligand was produced by the cells. We assumed an endosomal volume of 10 fL and endosomal surface area half that of the plasma membrane (Meyer et al., 2015). We assumed no fluid uptake of ligand and calculated the rate of change in endosomal ligand was derived by a mass balance of endosomal reactions. Endosomal ligand was assumed to completely sort into the lysosome from the endosome. All binding events were assumed to occur with 5-fold greater disassociation rate in the endosome due to its acidic pH (Fallon and Lauffenburger, 2000). Trafficking was therefore accounted for as:

$$\frac{dE}{dt}=-E\times k_{endo}+k_{\mathrm{rec}\;}\times \left(1-f_{\mathrm{sort}\;}\right)\times l\times \varphi$$

$$\frac{dl}{dt}=\frac{E\times k_{\mathrm{endo}\;}}{\varphi }-k_{rec}\times \left(1-f_{\mathrm{sort}\;}\right)\times l-k_{\deg\;}\times f_{\mathrm{sort}\;}\times l$$

where *E* and *I* indicate the abundance of the intracellular and extracellular forms, respectively. *φ* is the fractional membrane area of the endosomal compartment scaled to that of the surface membrane, and was assumed to be 0.5.

Free receptors and complexes were measured in units of number per cell and soluble ligands were measured in units of concentration (nM). Due to these unit choices for our species, the rate constants for ligand binding to free receptors had units of nM^−1^ min^−1^. Rate constants for the forward dimerization of free receptor to complex had units of cell min^−1^ number^−1^. Dissociation rates had units of min^−1^. All ligand-receptor binding processes had an assumed forward rate (k_bnd_) of 10^7^ M^−1^ sec^−1^. All forward dimerization reaction rates were assumed to be identical, represented by k_fwd_. Reverse reaction rates were unique. Experimentally-derived affinities of 1.0 (Gonnord et al., 2018), 59 (Walsh, 2012), 0.1 (Renauld et al., 1992), and 0.07 nM (Gonnord et al., 2018) were used for IL-4, −7, −9, and −21 binding to their cognate private receptors, respectively. IL-2 and −15 were assumed to have affinities of 10 nM and 0.065 nM for their respective α chains (Dubois et al., 2002; Mortier et al., 2006; Rickert et al., 2004), and affinities of 144 nM and 438 nM for their respective β-chains (Rickert et al., 2004). Rates k_5,rev_, k_10,rev_, and k_11,rev_ were set to their experimentally determined disassociation constants of 1.5, 12, and 63 min^−1^ (Rickert et al., 2004). Below are the ODEs pertaining to IL-2 binding and unbinding events, where L, α, and β signify IL-2, IL-2Rα, IL-2Rβ respectively:

$$\frac{d\alpha }{dt}=-k_{\mathrm{fbnd}\;}\alpha L+k_{1,\mathrm{rev}\;}[L\cdot \alpha ]+k_{8,\mathrm{rev}\;}\left[L\cdot \alpha \cdot \beta \cdot \gamma_{c}\right]-k_{\mathrm{fwd}\;}\times \left(\alpha [L\cdot \beta ]+a\left[L\cdot \beta \cdot \gamma_{c}\right]\right)+k_{12,\mathrm{rev}\;}[L\cdot \alpha \cdot \beta ]$$

$$\frac{d\beta }{dt}=-k_{\mathrm{fbnd}\;}\beta L+k_{2,\mathrm{rev}\;}[L\cdot \beta ]+k_{9,\mathrm{rev}\;}\left[L\cdot \alpha \cdot \beta \cdot \gamma_{c}\right]-k_{\mathrm{fwd}\;}\left(\beta [L\cdot \alpha ]+\beta \left[L\cdot \alpha \cdot \gamma_{c}\right]\right)+k_{11,\mathrm{rev}\;}[L\cdot \alpha \cdot \beta ]$$

$$\frac{d\gamma_{c}}{dt}=-k_{\mathrm{fwd}\;}\left([L\cdot \beta ]\gamma_{c}+[L\cdot \alpha ]\gamma_{c}+[L\cdot \alpha \cdot \beta ]\gamma_{c}\right)+k_{5,\mathrm{rev}\;}\left[L\cdot \beta \cdot \gamma_{c}\right]+k_{4,\mathrm{rev}\;}\left[L\cdot \alpha \cdot \gamma_{c}\right]+k_{10,\mathrm{rev}\;}\left[L\cdot \alpha \cdot \beta \cdot \gamma_{c}\right]$$

$$\frac{d[L\cdot \alpha ]}{dt}=-k_{\mathrm{fwd}\;}\left([L\cdot \alpha ]\beta +[L\cdot \alpha ]\gamma_{c}\right)+k_{11,\mathrm{rev}\;}[L\cdot \alpha \cdot \beta ]+k_{4,\mathrm{rev}\;}\left[2\cdot \alpha \cdot \gamma_{c}\right]+k_{\mathrm{fbnd}\;}L\alpha -k_{1,re\text{v}\;}[L\cdot \alpha ]$$

$$\frac{d[L\cdot \beta ]}{dt}=-k_{\mathrm{fwd}\;}\left([L\cdot \beta ]\alpha +[L\cdot \beta ]\gamma_{c}\right)+k_{12,\mathrm{rev}\;}[L\cdot \alpha \cdot \beta ]+k_{5,\mathrm{rev}\;}\left[L\cdot \beta \cdot \gamma_{c}\right]+k_{\mathrm{fbnd}\;}L\beta -k_{2,\mathrm{rev}\;}[L\cdot \beta ]$$

$$\frac{d[L\cdot \alpha \cdot \beta ]}{dt}=k_{\mathrm{fwd}\;}\left([L\cdot \beta ]\alpha +[L\cdot \alpha ]\beta -[L\cdot \alpha \cdot \beta ]\gamma_{c}\right)+k_{10,\mathrm{rev}\;}\left[L\cdot \alpha \cdot \beta \cdot \gamma_{c}\right]-k_{11,\mathrm{rev}\;}[L\cdot \alpha \cdot \beta ]-k_{12,\mathrm{rev}\;}[L\cdot \alpha \cdot \beta ]$$

$$\frac{d\left[L\cdot \alpha \cdot \gamma_{c}\right]}{dt}=-k_{9,rev}\left[L\cdot \alpha \cdot \beta \cdot \gamma_{c}\right]+k_{\mathrm{fwd}\;}\left([L\cdot \alpha ]\gamma_{c}-\left[L\cdot \alpha \cdot \gamma_{c}\right]\beta \right)-k_{4,rev}\left[L\cdot \alpha \cdot \gamma_{c}\right]$$

$$\frac{d\left[L\cdot \beta \cdot \gamma_{c}\right]}{dt}=k_{8,rev}\left[L\cdot \alpha \cdot \beta \cdot \gamma_{c}\right]+k_{\mathrm{fwd}\;}\left([L\cdot \beta ]\gamma_{c}-\left[L\cdot \beta \cdot \gamma_{c}\right]\alpha \right)-k_{5,rev}\left[L\cdot \beta \cdot \gamma_{c}\right]$$

$$\frac{d\left[L\cdot \alpha \cdot \beta \cdot \gamma_{c}\right]}{dt}=k_{\mathrm{fwd}\;}\left(\left[L\cdot \beta \cdot \gamma_{c}\right]\alpha +\left[L\cdot \alpha \cdot \gamma_{c}\right]\beta +[L\cdot \alpha \cdot \beta ]\gamma_{c}\right)-(k_{8,rev}+k_{9,rev}+k_{10,\mathrm{rev}}\text{)}\;\left[L\cdot \alpha \cdot \beta \cdot \gamma_{c}\right]$$

All above reactions also occur for IL-15, where L, α, and β signify IL-15, IL-2Rα, and IL-2Rβ respectively, and reverse rate parameters are substituted according to Table 1. The ODEs for IL-4 and IL-7 are derived by setting the abundance of α to 0, β representing the private receptor, and L representing the ligand concentration. Table 1 again lists the corresponding rate constants.

Initial values were calculated by assuming steady state in the absence of ligand. Differential equation solving was performed using the SUNDIALS solvers in C++, with a Python interface for all other code (Hindmarsh et al., 2005). Model sensitivities were calculated using the adjoint solution (Cao et al., 2002). Calculating the adjoint requires the partial derivatives of the differential equations both with respect to the species and unknown parameters. Constructing these can be tedious and error prone. Therefore, we calculated these algorithmically using forward-pass autodifferentiation implemented in Adept-2 (Hogan, 2017). A model and sensitivities tolerance of 10^−9^ and 10^−3^, respectively, were used throughout. We used unit tests for conservation of mass, equilibrium, and detailed balance to ensure model correctness.

#### Model fitting

We used Markov chain Monte Carlo to fit the unknown parameters in our model using previously published cytokine response data (Gonnord et al., 2018; Ring et al., 2012). Experimental measurements include pSTAT activity under stimulation with varying concentrations of IL-2, −15, −4, and −7 as well as time-course measurements of surface IL-2Rβ upon IL-2 and −15 stimulation. YT-1 human NK cells were used for all datasets involving IL-2 and IL-15. Human PBMC-derived CD4^+^TCR^+^CCR7^high^ cells were used for all IL-4 and −7 response data. All YT-1 cell experiments were performed both with the wild-type cell line, lacking IL-2Rα, and cells sorted for expression of the receptor. Data from Ring et al. (2012) and Gonnord et al. (2018) can be found in Figure 5 and Figure S3 of each paper, respectively. Measurements of receptor counts at steady state in Gonnord et al. (2018) were used to solve for IL-7Rα, IL-4Rα, and γ_c_ expression rates in human PBMCs.

Fitting was performed with the Python package PyMC3 (Salvatier et al., 2016). All unknown rate parameters were assumed to have a lognormal distribution with a standard deviation of 0.1; the only exception to these distributions was f_sort_ which was assumed to have a beta distribution with shape parameters of α = 20 and β = 40. Executing this fitting process yielded a distribution of each unknown parameter and a sum of squared error between model prediction and experimental data. The Geweke criterion metric was used to verify fitting convergence for all versions of the model (Figure S2; Geweke, 1992).

#### Tensor generation and factorization

To perform tensor factorization, we generated a three- (time points × cell types × ligand) or four-dimensional (time points × cell types × concentration × mutein) data tensor of predicted or measured ligand-induced signaling. Before decomposition, the tensor was variance scaled across each cell population. Tensor decomposition was performed using the Python package TensorLy (Kossaifi et al., 2019). Except where indicated otherwise, tensor decomposition was performed using non-negative canonical polyadic decomposition. Where indicated, non-negative Tucker decomposition was used.

#### Receptor abundance quantitation

Cryopreserved PBMCs (ATCC, PCS-800–011, Lot #81115172) were thawed to room temperature and slowly diluted with 9 mL prewarmed RPMI-1640 medium (GIBCO, 11875–093) supplemented with 10% fetal bovine serum (FBS, Seradigm, 1500–500, Lot #322B15). Media was removed, and cells washed once more with 10 mL warm RPMI-1640 + 10% FBS. Cells were brought to 1.5×10^6^ cells/mL, distributed at 250,000 cells per well in a 96-well V-bottom plate, and allowed to recover 2 hr at 37°C in an incubator at 5% CO2. Cells were then washed twice with PBS + 0.1% BSA (PBSA, GIBCO, 15260–037, Lot #2000843) and suspended in 50 μL PBSA + 10% FBS for 10 min on ice to reduce background binding to IgG.

Antibodies were diluted in PBSA + 10% FBS and cells were stained for 1 hr at 4°C in darkness with a gating panel (Panel 1, Panel 2, Panel 3, or Panel 4) and one anti-receptor antibody, or an equal concentration of matched isotype/fluorochrome control antibody. Stain for CD25 was included in Panel 1 when CD122, CD132, CD127, or CD215 was being measured (CD25 is used to separate T_reg_s from other CD4+ T cells).

Compensation beads (Simply Cellular Compensation Standard, Bangs Labs, 550, Lot #12970) and quantitation standards (Quantum Simply Cellular anti-Mouse IgG or anti-Rat IgG, Bangs Labs, 815, Lot #13895, 817, Lot #13294) were prepared for compensation and standard curve. One well was prepared for each fluorophore with 2 μL antibody in 50 μL PBSA and the corresponding beads. Bead standards were incubated for 1 hr at room temperature in the dark.

Both beads and cells were washed twice with PBSA. Cells were suspended in 120 μL per well PBSA, and beads to 50 μL, and analyzed using an IntelliCyt iQue Screener PLUS with VBR configuration (Sartorius) with a sip time of 35 and 30 s for cells and beads, respectively. Antibody number was calculated from fluorescence intensity by subtracting isotype control values from matched receptor stains and calibrated using the two lowest binding quantitation standards. T_reg_ cells could not be gated in the absence of CD25, so CD4+ T cells were used as the isotype control to measure CD25 in T_reg_ populations. Cells were gated (Figure S3), and then measurements were performed using four independent staining procedures over two days. Separately, the analysis was performed with anti-receptor antibodies at 3x normal concentration to verify that receptor binding was saturated. Replicates were summarized by geometric mean.

#### pSTAT5 measurement in PBMCs

Human PBMCs were thawed, distributed across a 96-well plate, and allowed to recover as described above. IL-2 (R&D Systems, 202-IL-010), IL-2 muteins, or IL-15 (R&D Systems, 247-ILB-025) were diluted in RPMI-1640 without FBS and added to the indicated concentrations. To measure pSTAT5, media was removed, and cells fixed in 100 μL of 10% formalin (Fisher Scientific, SF100–4) for 15 mins at room temperature. Formalin was removed, cells were placed on ice, and cells were gently suspended in 50 μL of cold methanol (−30°C). Cells were stored overnight at −30°C. Cells were then washed twice with PBSA, split into two identical plates, and stained 1 hr at room temperature in darkness using antibody panels 4 and 5 with 50 μL per well. Cells were suspended in 100 μL PBSA per well, and beads to 50 μL, and analyzed on an IntelliCyt iQue Screener PLUS with VBR configuration (Sartorius) using a sip time of 35 s and beads 30 s. Compensation was performed as above. Populations were gated (Figure S3), and the median pSTAT5 level extracted for each population in each well.

#### Recombinant proteins

IL-2/Fc fusion proteins were expressed using the Expi293 expression system according to manufacturer instructions (Thermo Scientific). Proteins were constructed as human IgG1 Fc fusions at the N- or C terminus to human IL-2 through a (G4S)4 linker. C-terminal fusions omitted the C-terminal lysine residue of human IgG1. The AviTag sequence GLNDIFEAQKIEWHE was included on whichever terminus did not contain IL-2. Fc mutations to prevent dimerization were introduced into the Fc sequence (Ishino et al., 2013). Proteins were purified using MabSelect resin (GE Healthcare). Proteins were biotinylated using BirA enzyme (BPS Biosciences) according to manufacturer instructions, and extensively buffer-exchanged into phosphate buffered saline (PBS) using Amicon 10 kDa spin concentrators (EMD Millipore). The sequence of IL-2Rβ/g Fc heterodimer was based on a reported active heterodimeric molecule (patent application US20150218260A1), with the addition of (G4S)2 linker between the Fc and each receptor ectodomain. The protein was expressed in the Expi293 system and purified on MabSelect resin as above. IL2-Rα ectodomain was produced with C-terminal 6xHis tag and purified on Nickel-NTA spin columns (QIAGEN) according to manufacturer instructions.

#### Octet binding assays

Binding affinity was measured on an Octet RED384 (ForteBio). Briefly, biotinylated monomeric IL-2/Fc fusion proteins were uniformly loaded to Streptavidin biosensors (ForteBio) at roughly 10% of saturation point and equilibrated for 10 mins in PBS + 0.1% bovine serum albumin (BSA). Association time was up to 40 mins in IL-2Rβ/g titrated in 2x steps from 400 nM to 6.25 nM, or IL-2Rα from 25 nM to 20 pM, followed by dissociation in PBS + 0.1% BSA. A zero-concentration control sensor was included in each measurement and used as a reference signal. Assays were performed in quadruplicate across two days. Binding to IL-2Rα did not fit to a simple binding model so equilibrium binding was used to determine the K_D_ within each assay. Binding to IL-2Rβ/γ fit a 1:1 binding model so on-rate (k_on_), off-rate (k_off_) and K_D_ were determined by fitting to the entire binding curve. Kinetic parameters and K_D_ were calculated for each assay by averaging all concentrations with detectable binding signal (typically 12.5 nM and above).

### QUANTIFICATION AND STATISTICAL ANALYSIS

For each figure, descriptions of pertinent statistical analyses or metrics used, the number of replicates of experiments performed, and the values of confidence intervals can be found in its corresponding figure caption. n indicates the number of times a particular experiment was performed (duplicate, triplicate, etc.) within each figure. All experiments performed using either YT-1 NK cells or hPBMCs were conducted using entirely separate experimental replicates gathered from a single cell line or donor, respectively.

The confidence intervals of model predictions were generated by using 100 draws from the Markov chain generated during the model fitting process to make 100 corresponding dose response predictions. The 10%–90% confidence interval indicates the range from the 10^th^ to 90^th^ percentile of the predicted signaling response magnitude.

For all quantification of cellular species abundances, whether pSTAT5 or receptor amounts, the mean fluorescent intensity (MFI) of flow cytometry data was calculated to determine population-level species abundance.

Experimental and predicted EC_50_s were estimated by fitting a standard Hill function to the dose-response curves using unbounded non-linear least-squares (Figure 4).

## Supplementary Material

1

2

3

## Figures and Tables

**Table 1.** Cytokine reverse binding constants

Rate/Role Description	IL-2	IL-15	IL-4	IL-7

α receptor	IL-2Rα	IL-15Rα	–	–
β receptor	IL-2Rβ	IL-2Rβ	IL-4Rα	IL-7Rα
Rate of ligand dissociation from α receptor	*k*_1,*rev*_	*k*_13,*rev*_	*k*_32,*rev*_	*k*_25,*rev*_
Rate of ligand dissociation from β receptor	*k*_2,*rev*_	*k*_14,*rev*_	–	–
Rate of γ_c_ dissociation from ligand · α · γ_c_ complex	*k*_4,*rev*_	*k*_16,*rev*_	*k*_33,*rev*_	*k*_27,*rev*_
Rate of γ_c_ dissociation from ligand · β · γ_c_ complex	*k*_5,*rev*_	*k*_17,*rev*_	–	–
Rate of α dissociation from ligand · α · β · γ_c_ complex	*k*_8,*rev*_	*k*_20,*rev*_	–	–
Rate of β dissociation from ligand · α · β · γ_c_ complex	*k*_9,*rev*_	*k*_21,*rev*_	–	–
Rate of γ_c_ dissociation from ligand · α · β · γ_c_ complex	*k*_10,*rev*_	*k*_22,*rev*_	–	–
Rate of β dissociation from ligand · α · β complex	*k*_11,*rev*_	*k*_23,*rev*_	–	–
Rate of α dissociation from ligand · α · β complex	*k*_12,*rev*_	*k*_24,*rev*_	–	–

**KEY RESOURCES TABLE:** 

REAGENT or RESOURCE	SOURCE	IDENTIFIER

Antibodies		

Anti-CD25, Brilliant Violet 421	BioLegend	Cat #356114; Clone #M-A251; RRID: AB_2562164
Anti-CD122, PE/Cy7	BioLegend	Cat #339014; Clone #TU27; RRID: AB_2562597
Anti-CD132, APC	BioLegend	Cat #338607; Clone #TUGh4; RRID: AB_2123585
Anti-CD215 1st mAb, APC	BioLegend	Cat #330210; Clone #JM7A4; RRID: AB_2561440
Anti-CD215 2nd mAb, APC	R&D Systems	Cat #FAB1471A; Clone #151303; RRID: AB_10890735
Anti-CD127, Alexa Fluor 488	BioLegend	Cat #351313; Clone #A019D5; RRID: AB_10895911
Anti-Ms IgG1κ, Brilliant Violet 421	BioLegend	Cat #400158; Clone #MOPC-21; RRID: AB_11150232
Anti-Md IgG1κ, PE/Cy7	BioLegend	Cat #400126; Clone #MOPC-21; RRID: AB_326448
Anti-Rat IgG2Bκ, APC	BioLegend	Cat #400612; Clone #RTK4530; RRID: AB_326556
Anti-Ms IgG2Bκ, APC	BioLegend	Cat #400320; Clone #MPC-11
Anti-Ms IgG2B, APC	R&D Systems	Cat #IC0041A; RRID: AB_357246
Anti-Ms IgG1κ, Alexa Fluor 488	BioLegend	Cat #400129; Clone #MOPC-21; RRID: AB_2890263
Anti-CD3, Brilliant Violet 605	BioLegend	Cat #300460; Clone #UCHT1; RRID: AB_2564380
Anti-CD8, Brilliant Violet 785	BioLegend	Cat #301046; Clone #RPA-T8; RRID: AB_2563264
Anti-CD45RA, PE/Dazzle 594	BioLegend	Cat #304146; Clone #HI100; RRID: AB_2564079
Anti-CD4, Brilliant Violet 785	BioLegend	Cat #300554; Clone #RPA-T4; RRID: AB_2564382
Anti-CD56, PE/Cy7	BioLegend	Cat #362510; Clone #5.1H11; RRID: AB_2563927
Anti-CD8, Alexa Fluor 647	BioLegend	Cat #301062; Clone #RPA-T8; RRID: AB_2564166
Anti-Foxp3, Alexa Fluor 488	BioLegend	Cat #320212; Clone #259D; RRID: AB_430887
Anti-CD4, Brilliant Violet 605	BioLegend	Cat #344646; Clone #SK3; RRID: AB_2734348
Anti-pSTAT5, Alexa Fluor 647	Cell Signaling Technology	Cat #9365; Clone #C71E5; RRID: AB_1904151
Anti-CD56, Alexa Fluor 488	BioLegend	Cat #362518; Clone #5.1H11; RRID: AB_2564093
Anti-pSTAT5, PE	Cell Signaling Technology	Cat #14603; Clone #D4737; RRID: AB_2798533

Chemicals, peptides, and recombinant proteins		

Simply Cellular Compensation Standard Beads	Bangs Labs	Cat #550
Quantum Simply Cellular anti-Mouse IgG	Bangs Labs	Cat #815
Quantum Simply Cellular anti-Rat IgG	Bangs Labs	Cat #817
MabSelect Resin	GE Healthcare	Cat #17519901
BirA enzyme	BPS Biosciences	Cat #70030
Interleukin-2 (IL-2)	R&D Systems	Cat #202-IL-010
Interleukin-15 (IL-15)	R&D Systems	Cat #247-ILB-025
Interleukin-2 muteins	This Paper	N/A

Critical commercial assays		

Octet RED384 Biolayer Interferometer	ForteBio	N/A

Deposited data		

All raw and processed cellular response data	This paper; Gonnord et al., 2018; Ring et al., 2012	https://github.com/meyer-lab/gc-cytokines

Experimental models: Cell lines		

Cryopreserved PBMCs	ATCC	Cat #PCS-800-011
Expi293F Cells	ThermoFisher Scientific	Cat #A14527

Software and algorithms		

Python3	Python Software Foundation	https://python.org/
C++	Standard C++ Foundation	https://isocpp.org/
SUNDIALS	Hindmarsh et al., 2005	https://computing.llnl.gov/projects/sundials
PyMC3	Salvatier et al., 2016	https://docs.pymc.io/
Adept-2	Hogan, 2017	https://github.com/rjhogan/Adept-2
TensorLy	Kossaifi et al., 2019	https://github.com/tensorly/tensorly
γ_c_ Mechanistic Binding Model	This paper	https://github.com/meyer-lab/gc-cytokines

## Highlights

- A dynamical model of the common γ-chain cytokines accurately predicts response
- Receptor trafficking is necessary for predicting ligand response in new contexts
- Tensor factorization maps responses across cell populations, receptors, and cytokines
- Pathway model provides design criteria for ligands with greater cell type selectivity
