# Supplementary material for: Modeling cell-specific dynamics and regulation of the common gamma chain cytokines
Source: Cell Rep. Author manuscript; Available in PMC 2021 Jun 5. (PMC8179794; doi:10.1016/j.celrep.2021.109044)
Supplement: 1 [file NIHMS1708599-supplement-1.pdf]

Cell Reports, Volume *35*

Supplemental information

Modeling cell-specific dynamics and regulation of the common gamma chain cytokines

Ali M. Farhat, Adam C. Weiner, Cori Posner, Zoe S. Kim, Brian Orcutt-Jahns, Scott M. Carlson, and Aaron S. Meyer

Supplement

Table S1: **Antibodies used to quantify receptors and cell types. Related to Figure 3.** *Panel 0:* Antibodies for IL-2, IL-15, and IL-7 receptor analysis; *Panel 1:* Antibodies to gate Naïve and Memory T-regulatory and T-helper cells; *Panel 2:* Antibodies to gate NK and CD56bright NK cells; *Panel 3:* Antibodies to gate Naïve and Memory Cytotoxic T cells; *Panel 4:* Antibodies to gate Naïve and Memory T-regulatory, T helper, and Cytotoxic cells, and NK cells for CD127 (IL-7) Quantitation; *Panel 5:* Antibodies to gate Memory and Naïve T-regulatory cells, Memory and Naïve T-helper cells; *Panel 6:* Antibodies to gate NK cells, CD56bright NK cells, and Cytotoxic T cells. CST: Cell Signaling Technology.

Antibody (clone)	Dilution	Fluorophore	Vendor (CAT#)	Panel
CD25 (M-A251)	1:120	Brilliant Violet 421	BioLegend (356114)	0
CD122 (TU27)	1:120	PE/Cy7	BioLegend (339014)	0
CD132 (TUGh4)	1:120	APC	BioLegend (3386)	0
CD215 1st mAb (JM7A4)	1:120	APC	BioLegend (330210)	0
CD215 2nd mAb (151303)	3:100	APC	R&D Systems (FAB1471A)	0
CD127 (A019D5)	1:120	Alexa Fluor 488	BioLegend (351313)	0
Ms IgG1κ (MOPC-21)	1:240	Brilliant Violet 421	BioLegend (400158)	0
Md IgG1κ (MOPC-21)	1:240	PE/Cy7	BioLegend (400126)	0
Rat IgG2Bκ (RTK4530)	1:60	APC	BioLegend (400612)	0
Ms IgG2Bκ (MPC-11)	1:120	APC	BioLegend (400320)	0
Ms IgG2B (133303)	3:100	APC	R&D Systems (IC0041A)	0
Ms IgG1κ (MOPC-21)	1:120	Alexa Fluor 488	BioLegend (400129)	0
CD3 (UCHT1)	1:120	Brilliant Violet 605	BioLegend (300460)	1
CD4 (RPA-T4)	1:120	Brilliant Violet 785	BioLegend (300554)	1
CD127 (A019D5)	1:120	Alexa Fluor 488	BioLegend (351313)	1
CD45RA (HI100)	1:120	PE/Dazzle 594	BioLegend (304146)	1
CD3 (UCHT1)	1:120	Brilliant Violet 605	BioLegend (300460)	2
CD56 (5.1H11)	1:120	PE/Dazzle 594	BioLegend (362544)	2
CD3 (UCHT1)	1:120	Brilliant Violet 605	BioLegend (300460)	3
CD8 (RPA-T8)	1:120	Brilliant Violet 785	BioLegend (301046)	3
CD45RA (HI100)	1:120	PE/Dazzle 594	BioLegend (304146)	3
CD25 (M-A251)	1:120	Brilliant Violet 421	BioLegend (356114)	4
CD3 (UCHT1)	1:120	Brilliant Violet 605	BioLegend (300460)	4
CD4 (RPA-T4)	1:120	Brilliant Violet 785	BioLegend (300554)	4
CD127 (A019D5)	1:120	Alexa Fluor 488	BioLegend (351313)	4
CD45RA (HI100)	1:120	PE/Dazzle 594	BioLegend (304146)	4
CD56 (5.1H11)	1:120	PE/Cy7	BioLegend (362510)	4
CD8 (RPA-T8)	1:120	Alexa Fluor 647	BioLegend (301062)	4
Foxp3 (259D)	1:50	Alexa Fluor 488	BioLegend (320212)	5
CD25 (M-A251)	1:120	Brilliant Violet 421	BioLegend (356114)	5
CD4 (SK3)	1:120	Brilliant Violet 605	BioLegend (344646)	5
CD45RA (HI100)	1:120	PE/Dazzle 594	BioLegend (304146)	5
pSTAT5 (C71E5)	1:120	Alexa Fluor 647	CST (9365)	5
CD3 (UCHT1)	1:120	Brilliant Violet 605	BioLegend (300460)	6
CD8 (RPA-T8)	1:120	Alexa Fluor 647	BioLegend (301062)	6
CD56 (5.1H11)	1:120	Alexa Fluor 488	BioLegend (362518)	6
pSTAT5 (D4737)	1:120	PE	CST (14603)	6

Table S2: **Modified IL-2 ligands and their respective mutations, and Fc conjugations. Related to Figure 6.**

Ligand	Fc Conjugation	Specificity Mutation	Other Mutations
F42Q N-Term	N-Terminus	F42Q	V69A/Q74P/C125S
N88D C-term	C-Terminus	N88D	C125A
R38Q N-term	N-Terminus	R38Q	V69A/Q74P/C125S
V91K C-term	C-Terminus	V91K	C125A
WT C-term	C-Terminus	Wild-type	C125A
WT N-term	N-Terminus	Wild-type	V69A/Q74P/C125S

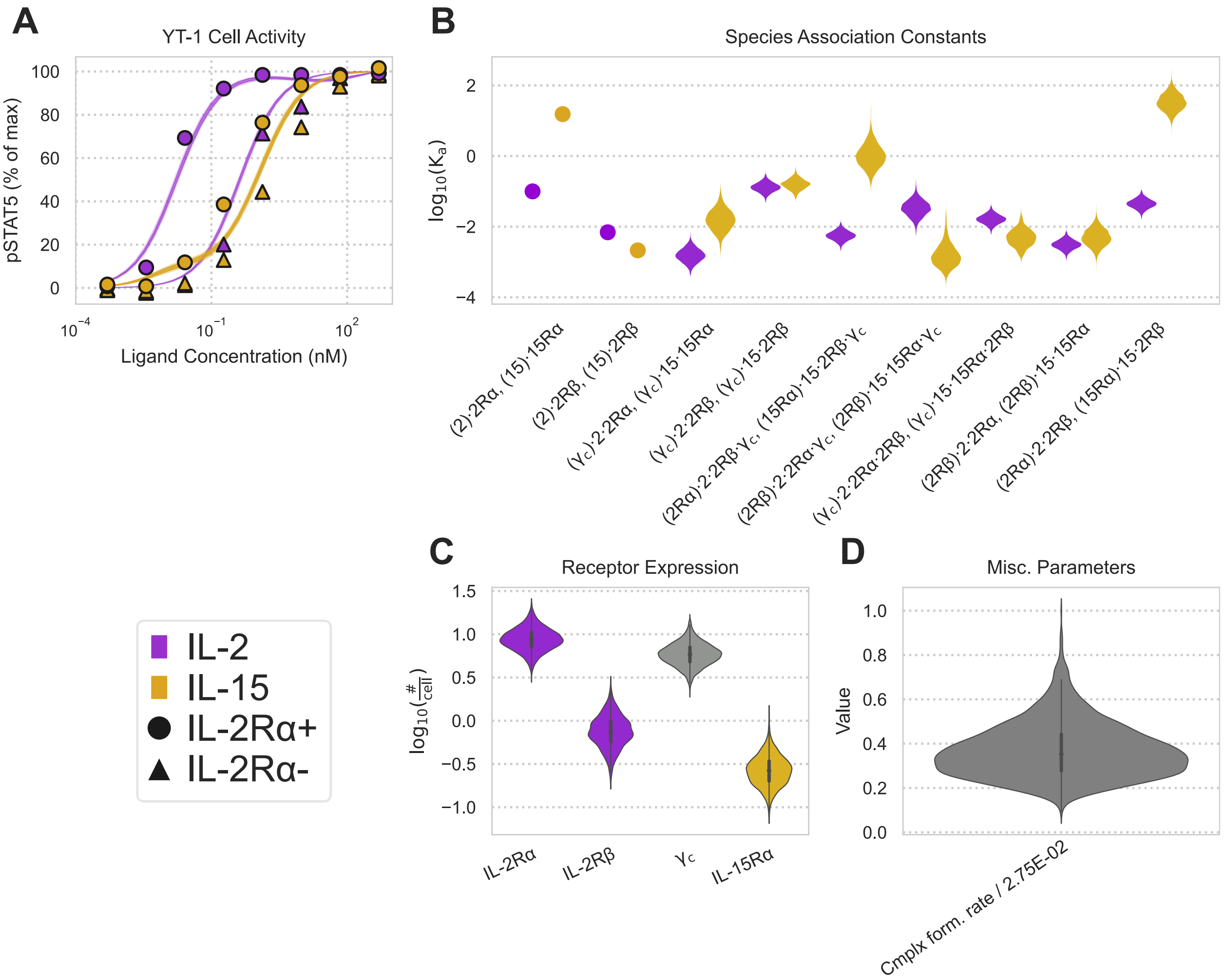

Figure S1: **Model without trafficking fitted to IL-2/-15 dose response. Related to Figure 1.** A) Model without trafficking fit to IL-2 and IL-15 pSTAT5 dose response data (Ring et al., 2012). This model was not fit to the surface IL-2Rβ measurements since no receptors were allowed to internalize from the cell surface (Fig. 1B-D). B) Posterior distributions for the analogous association constants of IL-2 and IL-15. Association constants measured in literature are represented by dots. Association constants are shown for species in parentheses complexing with following species. K_as for (2)·2Rα, (15)·15Rα, (2)·2Rβ, and (15)·2Rβ have units of nM, all other K_as have units of # $\times$ cell^{-1}. C) Posterior distributions for receptor surface abundance in no-trafficking model. D) Posterior distributions after data fitting for no-trafficking model. C_5, which is a constant in the sigmoidal relationship our model uses to translate active signaling complexes to pSTAT levels, has units of # $\times$ cell^{-1}, Complex Formation Rate (k_{fwd}) has units of cell $\times$ #$^{-1}$ $\times$ min^{-1}.

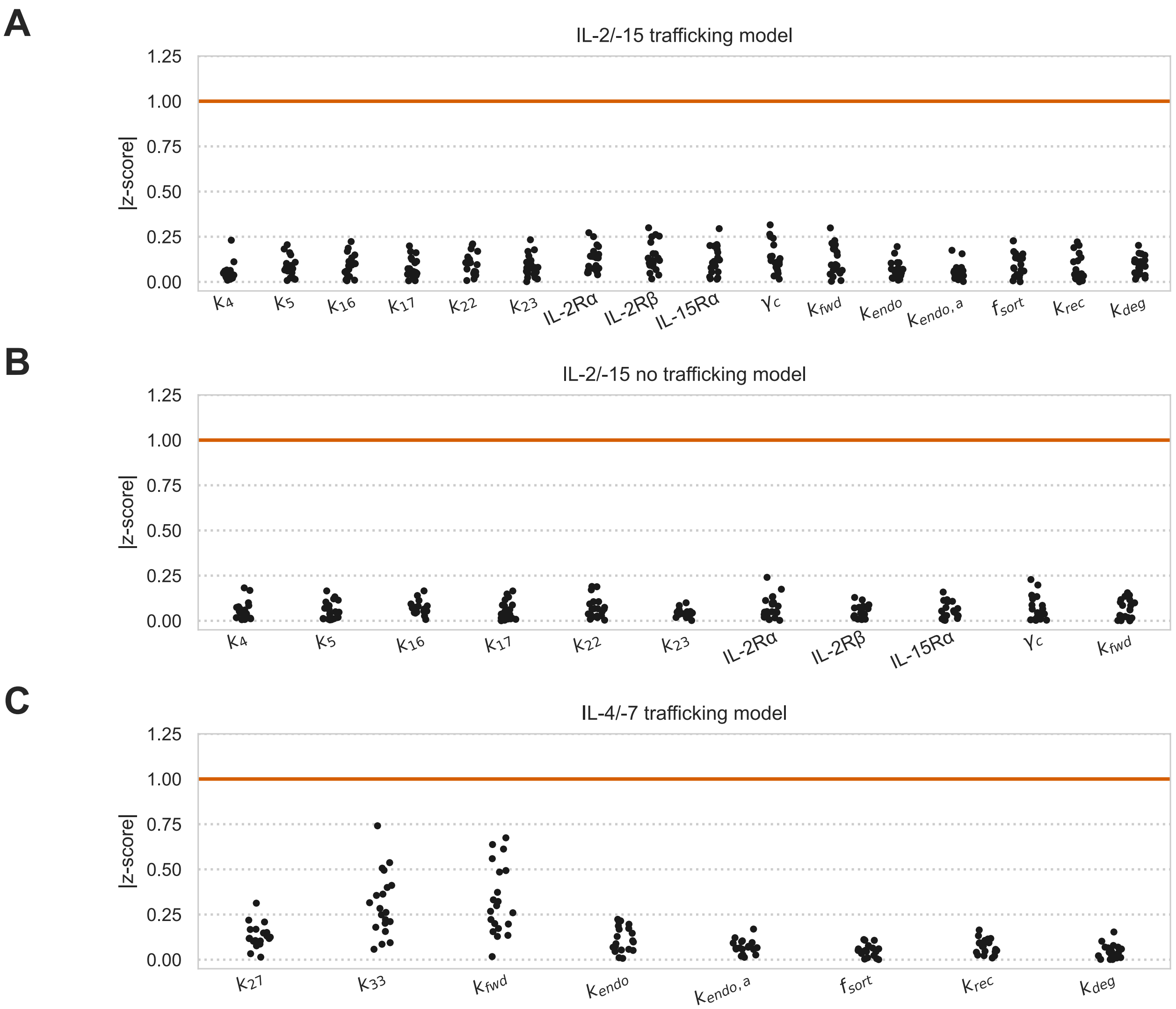

Figure S2: **Geweke criterion scores for model fitting with and without trafficking. Related to Figure 1, 2, 4, and 6.** Geweke criterion z-scores in all subplots were calculated using 20 intervals in the first 10% and last 50% of MCMC chain. Scores of $|z| < 1$ imply fitting convergence. A-B) IL-2/-15 with and without trafficking. C) IL-4/-7 with trafficking (Fig. S1).

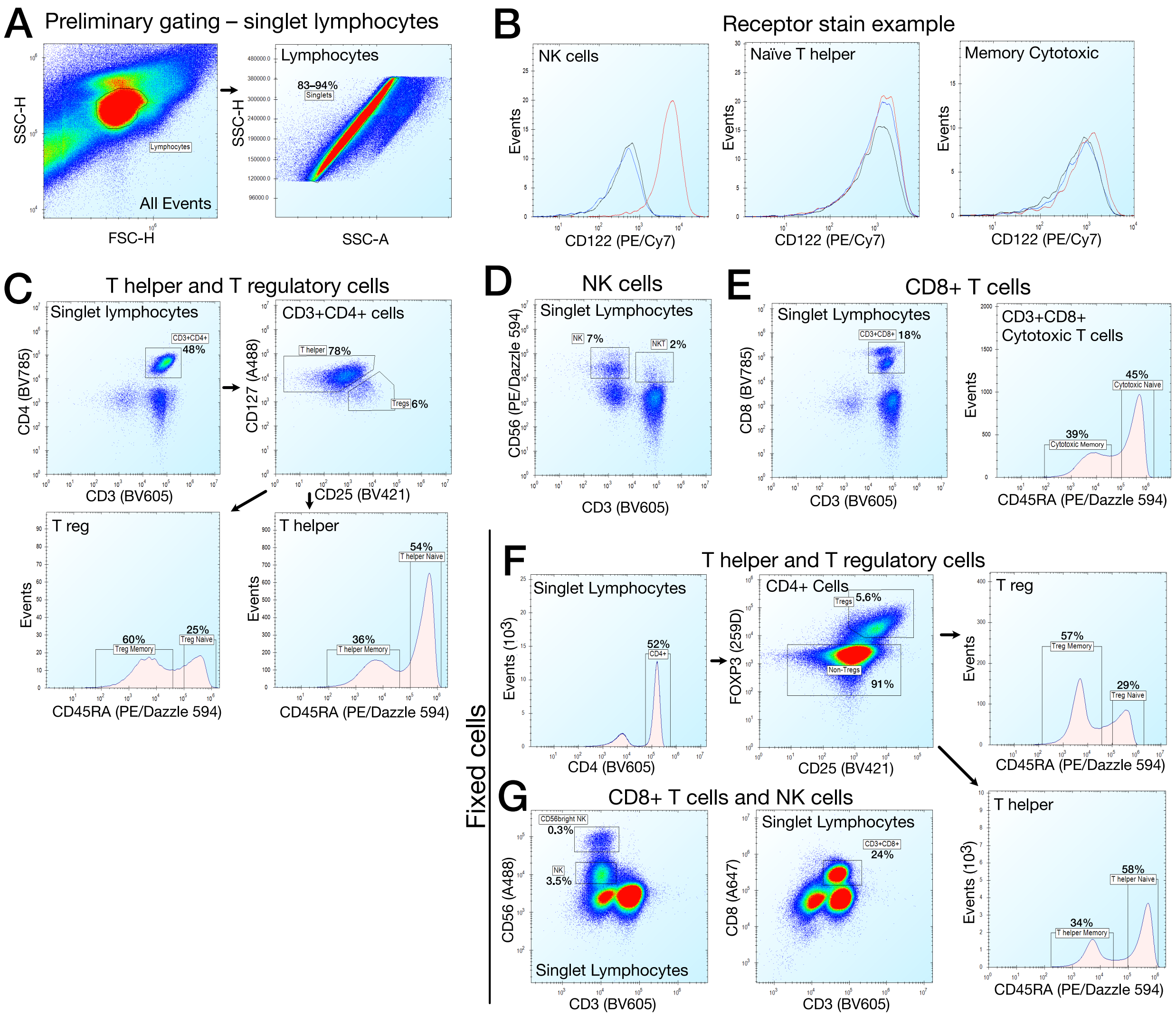

Figure S3: **Receptor quantification and gating of PBMC-derived immune cell types. Related to Figure 3.** A) Preliminary gating for single lyphocytes. B) Example staining for CD122 (red), the corresponding isotype control (blue), and unstained cells (black). C) Gating for live T helper and T regulatory cells during receptor quantification. D) Live cell NK cell gating. E) Live cell CD8+ T cell gating. F) Gating for fixed T helper and T regulatory cells during pSTAT5 quantification. G) Fixed CD8+ T cell and NK cell gating.

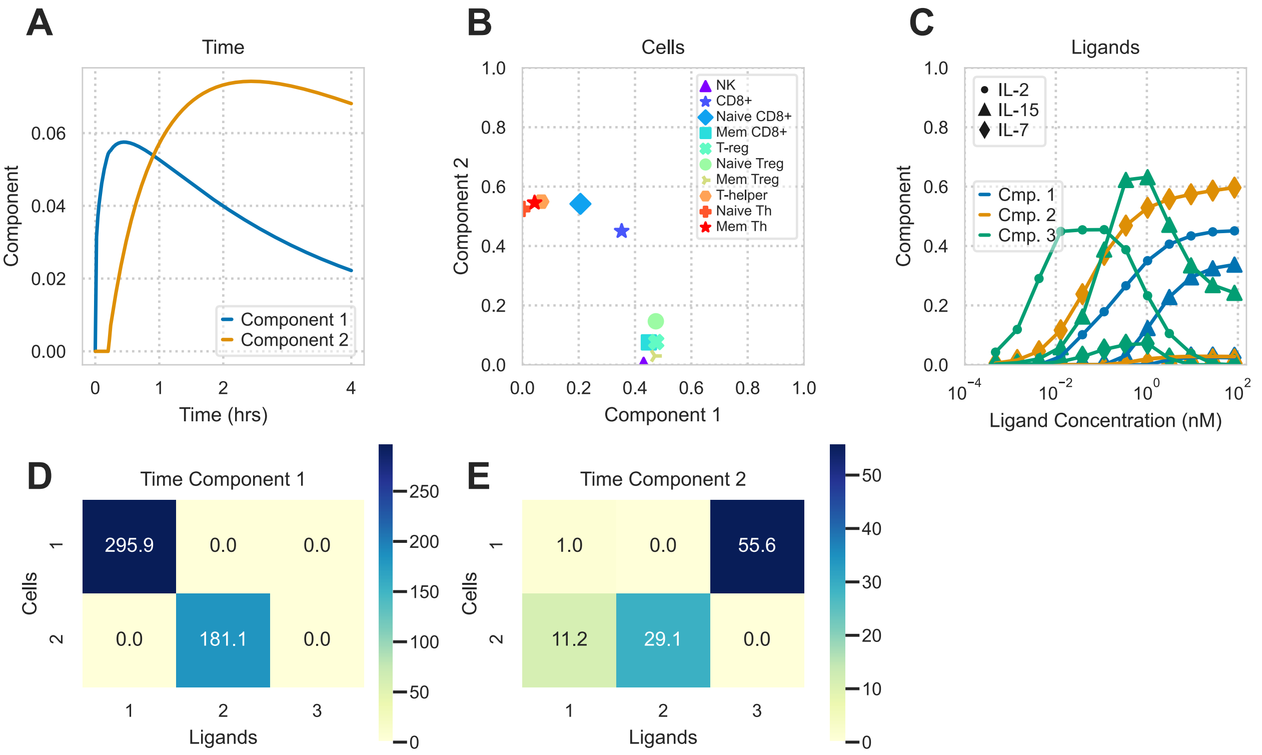

Figure S4: **Tucker factorization of predicted immune cell type responses. Related to Figure 5.** A) Timepoint decomposition plot showing factorization component values against time after decomposing the tensor's first dimension into 2 components. B) Decomposition plot along the second (cell) dimension after decomposing it to 2 components showing the ten cell type values along each component. C) Ligand decomposition plot along the tensor's third dimension after decomposing it into 3 components. D–E) Slices of the Tucker core tensor corresponding to time component 1 (D) and 2 (E).

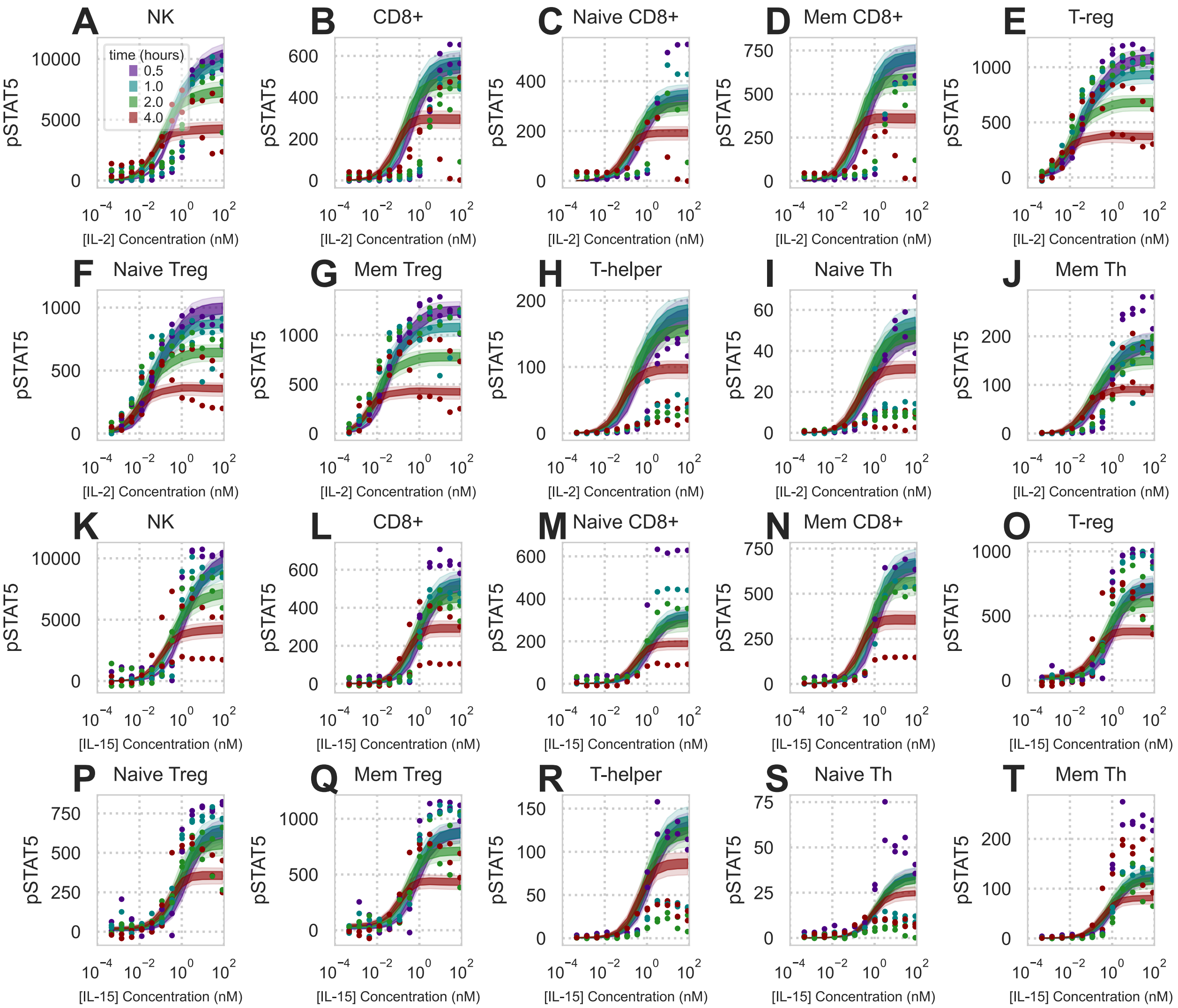

Figure S5: **Full panel of predicted versus actual immune cell type responses. Related to Figure 4.** Dots represent flow cytometry measurements and shaded regions represent 10-90% confidence interval for model predictions. Time of pSTAT5 activity measurement is denoted by color. All cell populations were stimulated with either IL-2 (A-J) or IL-15 (K-T). Experiments were performed in duplicate (N = 2).

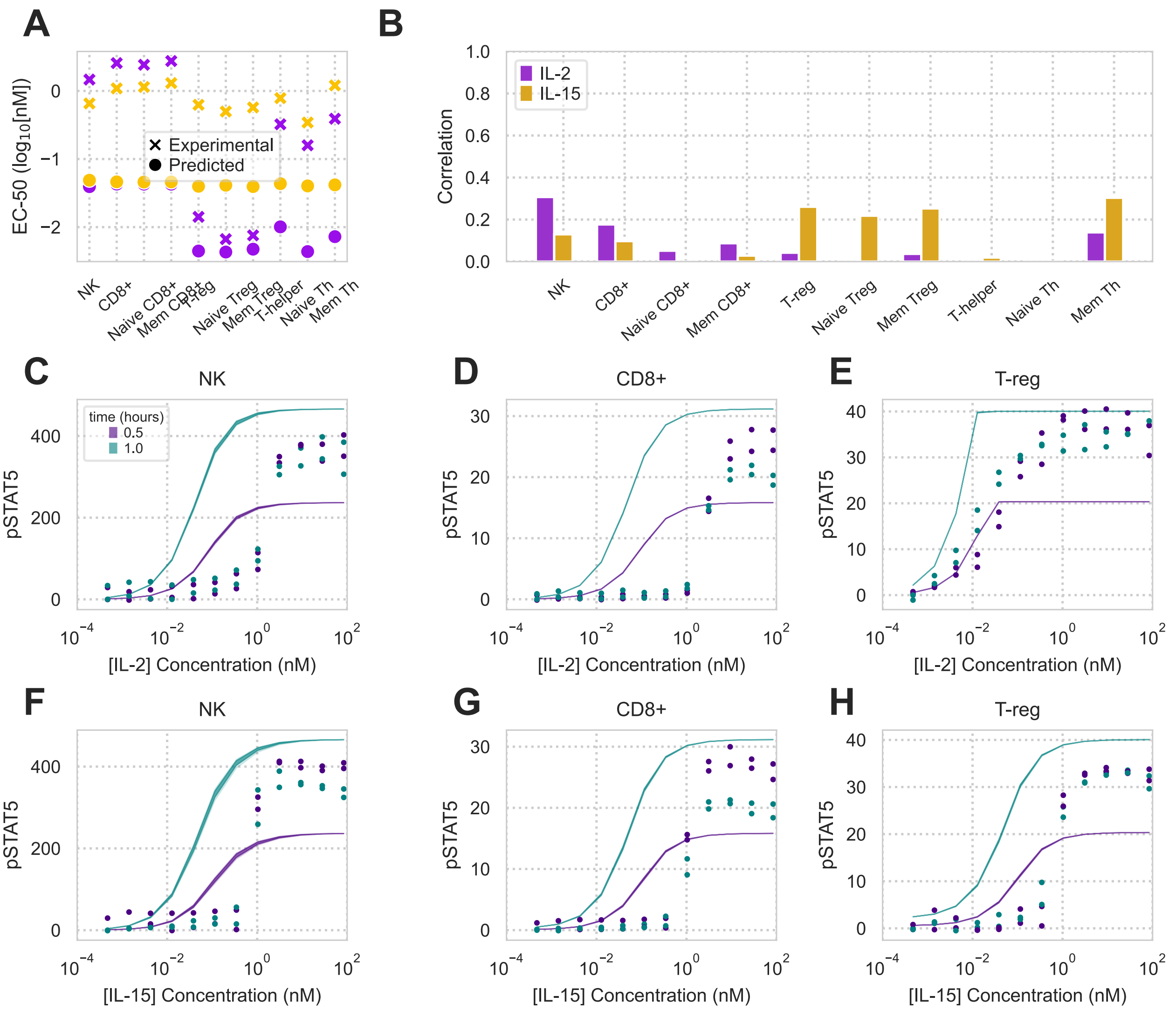

Figure S6: **Model without trafficking is unable to accurately predict PBMC signaling response. Related to Figure 4.** A) Both experimentally-derived and model without trafficking-predicted EC_{50}s of dose response across IL-2/-15 and all 10 cell types. EC_{50}s are shown for 1 hr time point. B) Pearson correlation coefficients between model without trafficking prediction and experimental measurements for all 10 cell populations. C–H) pSTAT5 response to IL-2 (C–E) or IL-15 (F–H) dose responses in NK, CD8+, and T_{reg} cells. Predictions were made using model without trafficking. Experiments were performed in duplicate (N = 2).

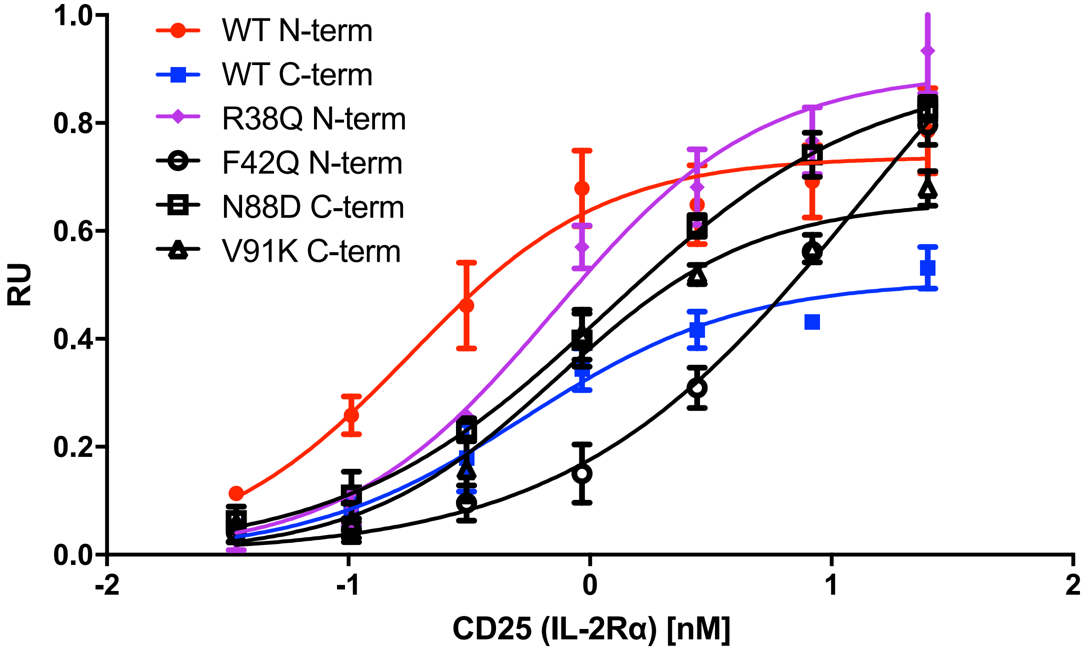

Figure S7: **Cytokine affinity measurements to IL-2Rα. Related to Figure 6.** Binding is quantified in relative units using biolayer interferometry.

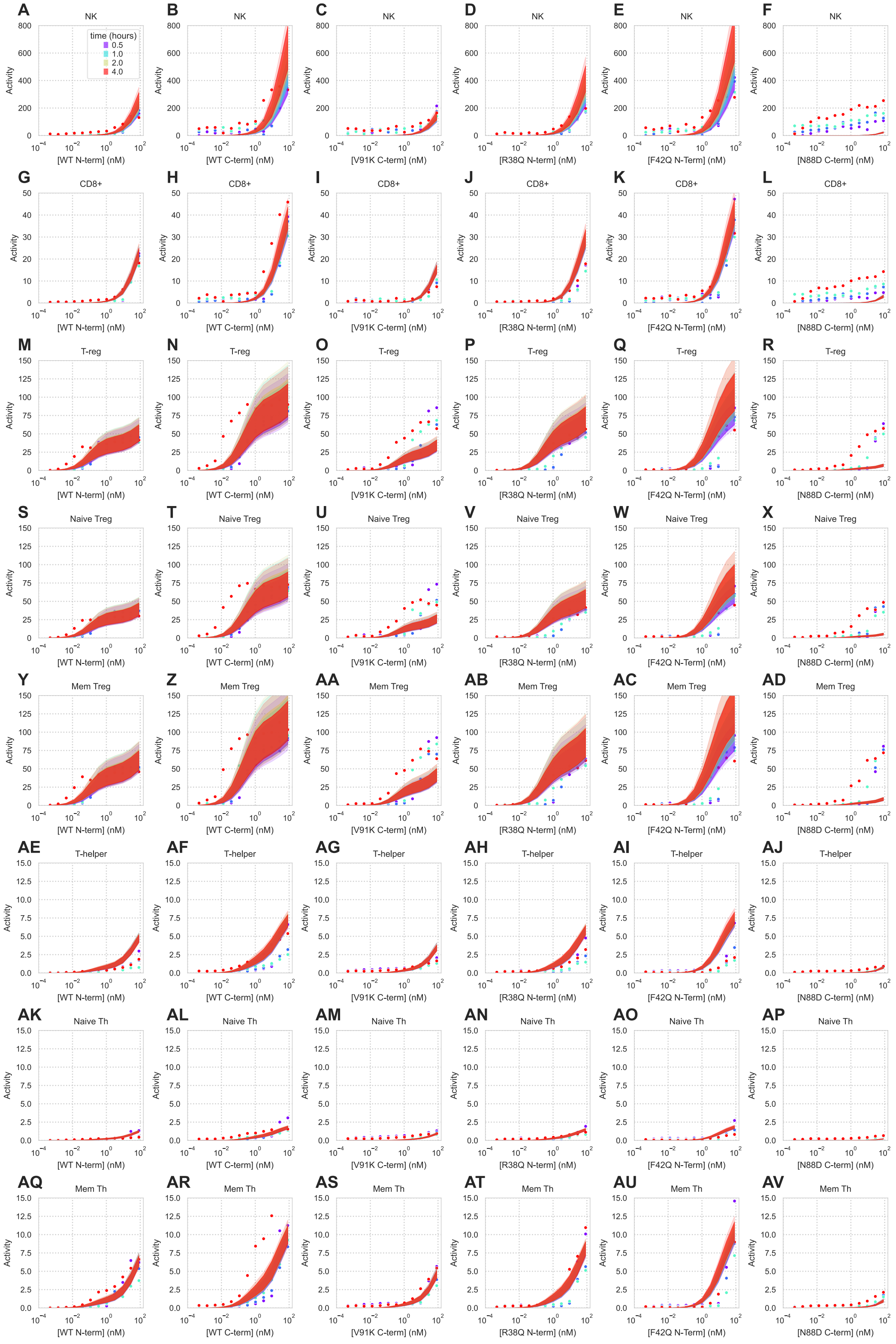

Figure S8: **Full panel of predicted versus actual immune cell type responses to IL-2 muteins. Related to Figure 6.** Dots represent flow cytometry measurements and shaded regions represent 10-90% confidence interval for model predictions. Time of pSTAT5 activity measurement is denoted by color. Cell populations were stimulated with IL-2 muteins of varying IL-2Rα and IL-2Rβ/γ_c binding affinities. Experiments were performed once (N = 1).
